# Synthesis, Characterization, and Docking Study of Novel Thioureidophosphonate-Incorporated Silver Nanocomposites as Potent Antibacterial Agents

**DOI:** 10.3390/pharmaceutics15061666

**Published:** 2023-06-06

**Authors:** Ahmed I. El-Tantawy, Elshaymaa I. Elmongy, Shimaa M. Elsaeed, Abdel Aleem H. Abdel Aleem, Reem Binsuwaidan, Wael H. Eisa, Ayah Usama Salman, Noura Elsayed Elharony, Nour F. Attia

**Affiliations:** 1 Department of Chemistry, Faculty of Science, Menoufia University, Shibin El Kom 32511, Egypt; aelgokha@yahoo.com (A.A.H.A.A.); nora7arony@yahoo.com (N.E.E.); 2 Department of Pharmaceutical Sciences, College of Pharmacy, Princess Nourah bint Abdulrahman University, P.O. Box 84428, Riyadh 11671, Saudi Arabia; rabinsuwaidan@pnu.edu.sa; 3 Department of Analysis and Evaluation, Egyptian Petroleum Research Institute, Cairo 11727, Egypt; shy_saeed@yahoo.com; 4 Spectroscopy Department, Physics Division, National Research Centre (NRC), Cairo 12622, Egypt; wael_karnor@yahoo.com; 5 Department of Botany and Microbiology, Faculty of Science, Menoufia University, Shibin El Kom 32511, Egypt; ayahsalman6060@gmail.com; 6 Gas Analysis and Fire Safety Laboratory, Chemistry Division, National Institute for Standards, 136, Giza 12211, Egypt; drnour2005@yahoo.com

**Keywords:** thioureidophosphonates, silver nanocomposites, antibacterial, bactericidal, antifouling, antibiofilm, molecular docking

## Abstract

Newly synthesized mono- and bis-thioureidophosphonate (MTP and BTP) analogues in eco-friendly conditions were employed as reducing/capping cores for 100, 500, and 1000 mg L^−1^ of silver nitrate. The physicochemical properties of silver nanocomposites (MTP(BTP)/Ag NCs) were fully elucidated using spectroscopic and microscopic tools. The antibacterial activity of the nanocomposites was screened against six multidrug-resistant pathogenic strains, comparable to ampicillin and ciprofloxacin commercial drugs. The antibacterial performance of BTP was more substantial than MTP, notably with the best minimum inhibitory concentration (MIC) of 0.0781 mg/mL towards *Bacillus subtilis*, *Salmonella typhi*, and *Pseudomonas aeruginosa*. Among all, BTP provided the clearest zone of inhibition (ZOI) of 35 ± 1.00 mm against *Salmonella typhi*. After the dispersion of silver nanoparticles (AgNPs), MTP/Ag NCs offered dose-dependently distinct advantages over the same nanoparticle with BTP; a more noteworthy decline by 4098 × MIC to 0.1525 × 10^−^^3^ mg/mL was recorded for MTP/Ag-1000 against *Pseudomonas aeruginosa* over BTP/Ag-1000. Towards methicillin-resistant *Staphylococcus aureus* (*MRSA*), the as-prepared MTP(BTP)/Ag-1000 displayed superior bactericidal ability in 8 h. Because of the anionic surface of MTP(BTP)/Ag-1000, they could effectively resist *MRSA* (ATCC-43300) attachment, achieving higher antifouling rates of 42.2 and 34.4% at most optimum dose (5 mg/mL), respectively. The tunable surface work function between MTP and AgNPs promoted the antibiofilm activity of MTP/Ag-1000 by 1.7 fold over BTP/Ag-1000. Lastly, the molecular docking studies affirmed the eminent binding affinity of BTP over MTP—besides the improved binding energy of MTP/Ag NC by 37.8%—towards *B. subtilis*-2FQT protein. Overall, this study indicates the immense potential of TP/Ag NCs as promising nanoscale antibacterial candidates.

## 1. Introduction

Recently, the emergence of antibiotic-resistant microbes has been seen as a global health concern affecting many people’s lives [1]. Regarding bacteria, the overuse of antibiotics, combined with poor health awareness and lack of technological capacity, has significantly increased the ability of bacteria to cross the boundaries of living systems, giving rise to more resistant genes, and raising the incidence of disease and death. Consequently, antimicrobial resistance (AMR) has caused several obstacles in public health care [1,2,3]. The growth of extracellular polymeric substances (EPS) as protective architectures surrounding bacteria is another sign of bacterial resistance [4]. Thus, finding novel, effective, and long-lasting biofilm preventing and disrupting agents is a key challenge for combating AMR [1,5]. Accordingly, the revival of nanotechnology along with the industrial expansion of antibiotics have lately pushed metal nanoparticles (MNPs) to the forefront of attention [6,7]. Noble MNPs have drawn utmost focus of researchers in several assorted domains with respect to optical [8], catalytic [9], energetic [10], and medical applications [11,12,13]. The unrivalled surface energy of noble MNPs promotes the generation of new superb physical, chemical, and biological properties such as small particle sizes, light susceptibility, tailored morphology and surface interactions, chemical stability, and biological compatibility [14,15]. Among noble MNPs, silver nanoparticles (AgNPs) surpassed all due to their tunable surface engineering, potent biological activity, multiple inhibition mechanisms, and facile methods of synthesis [16,17,18,19]. However, some demerits concerning the antibacterial activity of AgNPs were encountered, such as the possibility of particle agglomeration, elevated cost, developed AMR to AgNPs, and their toxicological effects at high concentrations [20,21]. Therefore, it was imperative to decorate AgNPs on inert surfaces [22] or/and combine them with active materials to avoid agglomeration and provide better antibacterial potency with less toxic effects of free AgNPs [20]. Therefore, alternative approaches are being pursued for the fabrication of AgNP composites by employing graphene [23], polymers [24], plant extract [16], clay [25], polysaccharide [26], and fatty acid [27]. The main disadvantages related to these nanohybrids were incomplete antibacterial efficacy [24], rapid release of active components [28], cost and time-ineffective operation [29], aggregation affinity [30], and higher cytotoxicity [31,32,33]. On top of this, α-aminophosphonates (α-APs), as a significant class of organophosphorus compounds, were remarkably exploited in several amplified domains [34,35]. Lately, α-APs have captivated researchers’ interests in numerous applicable dimensions such as wastewater treatment [36,37], rare earth metal removal [38,39], catalysis [40], sensing [41], flame retardancy [42], agrochemical technology [35,42], and corrosion inhibition [43]. Moreover, α-APs have had unprecedented success in several medical arenas, such as antioxidant, antifungal, anti-HIV agents, antimicrobial, anticancer, antiviral, peptidomimetics, and enzyme inhibitors [34,35,44]. This growing interest was ascribed to their structural analogy to α-amino acids, ease of synthesis, elevated metabolic and chemical stability, high atom economy, bioavailability, structural and functional diversity, insignificant cytotoxicity, and potent biological properties [34,35,45]. Therefore, α-APs were deemed a worthy choice for AgNPs dispersion to bring extra synergistic antibacterial properties as part of a sustainable approach. Remarkably, our group has long been involved in developing different series of phosphonate derivatives in medical applications as antibacterial agents [45,46], urokinase-type plasminogen inhibitors [47], DNA gyrase inhibitors [48], anticancer [49], antifungal agents [49]. This is in addition to designing and synthesizing other potent antibacterial agents for various industrial applications [22,50,51]. Based on the above considerations, two novel thioureidophosphonates (TPs) were synthesized via non-metal green catalyst and incorporated with silver ion precursor giving TP-wrapped silver nanocomposites (TP/Ag NCs). The main objective of the study was to explore the impact of functionalized AgNPs on the antibacterial activity raw TP analogues.

## 2. Materials and Methods

### 2.1. Characterization Techniques

The Fourier transform infrared spectroscopy (FT-IR) analysis has been accomplished for all samples on Bruker Alpha II spectrometer, Bremen, Germany, in the wavenumber range 4000–400 cm^−1^. The (^1^H-, ^13^C-, and ^31^P) nuclear magnetic resonance (NMR) spectra were consecutively recorded at 400, 101, and 162 MHz in DMSO-d_6_ using a Bruker Avance III HD, 600 MHz-NMR spectrometer probe, and BBFO cryoprobe (JEOL, Tokyo, Japan). The elemental analysis of samples (C, H, N, and S) was undertaken for the raw TP derivatives (MTP and BTP) by CHNS Vario EL III Elementar analyzer, Germany. The phosphorus content (%) was estimated via the chemical digestion in sulfuric/nitric solution and photometric measurements at λ_max_ = 410 nm. Melting points (m.p) were recorded using (DMP-600, A and E Lab, London, UK) without correction. The surface images of nanocomposites were obtained using transmission electron microscope (TEM) by JEOL, JEM-2100 Tokyo, Japan. The crystalline properties of nanocomposites were obtained using powder X-ray diffraction (XRD) analysis using Shimadzu 6000 X-ray diffractometer with Cu Kα-radiation (λ = 1.54 nm) at 25 °C in the 2θ range of 10–80° operating at a scan rate of 1 deg/min. X-ray photoelectron spectroscopy (XPS) was conducted using a Perkin Elmer PHI 5600, Perkin Elmer Instruments, Waltham, MA, USA, with analytical zone’s diameter of 1 mm and indium sheets for sample deposition using Al Kα X-rays radiation source (200 W). The photometric assays of antibacterial results were measured by (Infinite F50 Robotic absorbance microplate reader) in the wavelength range of 400 to 750 nm. The zeta potential charge of silver nanocomposites was determined via dynamic light scattering using Zetasizer Nano ZS, Malvern Instruments Ltd., Malvern, UK. Scanning electron microscopy (SEM) was performed using JSM 6390 LA, JEOL, Tokyo, Japan, with a 15 KV accelerating voltage.

### 2.2. Chemicals

Thiourea (99.0%, purity) and triphenyl phosphite (98.0%, purity) were obtained from Macklin (Beijing, China). Terephthalaldehyde (98.0%, purity), pyridinium trifluoromethanesulfonate (98.0%, purity) (as protic ionic liquid), and silver nitrate (99.8%, purity) (AgNO_3_) were purchased from Aladdin (Beijing, China). Solvents, such as acetonitrile (CH_3_CN), ethanol, and methanol were obtained from a commercial supplier (Sigma Aldrich, Cairo, Egypt) and used without further drying and purification. Deionized water was used to prepare all solutions and suspensions.

### 2.3. Preparation of α-Thioureidophosphonate Cores

The synthesis process included preparation of two different systems of mono and bis thioureidophosphonate (TP) products, where the involved components (thiourea, terephthalaldehyde, and triphenyl phosphite) were dissolved in acetonitrile (CH_3_CN, 5 and 10 mL) with the aid of a pyridinium trifluoromethanesulfonate (pyridinium triflate) as a Lewis acid protic ionic liquid-based catalyst. In the meantime, thiourea (1.42 g, 18.62 mmol)/(2.84 g, 37.24 mmol), (2.50 g, 18.61 mmol) terephthalaldehyde, triphenyl phosphite (5.78 g, 18.62 mmol)/(11.55 g, 37.24 mmol), and pyridinium triflate (0.21 g, 5 mole %)/(0.42 g, 5 mole %) in 5 and 10 mL of CH_3_CN were progressively incorporated, affording MTP and BTP in a molar ratio of 1:1:1 and 2:1:2, respectively. Further, both solutions were magnetically stirred overnight under ambient conditions and the reaction was monitored via qualitative thin layer chromatography (TLC) using hexane–methylene chloride (3:1) as eluent mixture. Eventually, the final products were filtered off under vacuum, washed with methanol, dried, and kept in a desiccator for 2 days giving MTP and BTP in good yields.

Diphenyl ((4-formylphenyl) (thioureido)methyl) phosphonate, denoted as mono-thioureidophosphonate (MTP):

Light-beige solid, yield: (7.25 g, 91.72%), m.p: 160–163 °C, FT-IR: 3457.74–3311.18 cm^−1^ (NH)str. + (NH_2_) str. overlapped, 3057.58 cm^−1^ (C=CH str., benzene ring), 2894.63 cm^−1^ (C H str., aliphatic), 1698.02 cm^−1^ (-CHO, formyl), 1590.99 cm^−1^, (NH+NH_2_) bending, 1525.42 cm^−1^ (>C=C<) phenyl rings, 1488.78 cm^−1^ (>C=S) asym., 1194.69 cm^−1^ (-P=O), 944.95 cm^−1^ (P-O-C), 760.78 cm^−1^ (P–C), and 684.61 cm^−1^ (>C=S) sym. ^1^H-NMR; δ ppm: 5.50 (d, 1H, P-C–H), 6.63–7.99 (m, Ar-H, 14H), 9.40–9.52 (br, 2H,-NH_2_), 9.76 (br. s, 1H, >NH), and 10.04 (s, 1H, -CHO). ^13^C-NMR (DMSO-d6, 101 MHz); δ ppm: 54.59 and 56.16 ppm (P-C–H), 115.24, 120.22, 120.53, 125.39, 127.96, 128.92, 129.90, 149.80 (Ar-C), 183.84 (>C=S), and 192.73 (-CHO). ^31^P-NMR (DMSO-d6, 162 MHz); δ 14.08 ppm. Elemental analysis calc. (%) for C_21_H_19_N_2_O_4_PS: C, 59.15; H, 4.49; N, 6.57; P, 7.26; S, 7.52; and O, 15.01; meas. (%): C, 57.85; H, 4.27; N, 6.34; P, 6.97; and S, 6.99.

Tetraphenyl (1,4-phenylenebis(thioureidomethylene)) bis(phosphonate) denoted as bis-thioureidophosphonate (BTP):

Light-yellow solid, yield: (11.75 g, 87.71%), m.p: 135–137 °C, FTIR: 3310.21 cm^−1^ [(NH)str. + (NH_2_) str. overlapped], 3057.58 cm^−1^ (C=CH str., benzene ring), 2895.59 cm^−1^ (C–H str., aliphatic), 1591.95 (>NH + -NH_2_) bending, 1527.35 cm^−1^ (>C=C<) phenyl rings, 1489.74 cm^−1^ (>C=S) asym., 1195.65 (-P=O), 947.84 (P-O-C), 761.74 cm^−1^ (P–C), and 761.74 cm^−1^ (>C=S) sym. ^1^H-NMR; δ ppm: 5.93 (s, 2H, P-C–H), 6.54–7.61 (Ar-H, m, 24H), 9.21–9.33 (br. m, 4H, NH_2_), and 9.38 ppm (br. s, 2H). ^13^C-NMR (DMSO- d6, 101 MHz); δ ppm: 54.50 and 56.06 (P-C–H), 115.26, 120.29, 120.86, 125.40, 127.50, 129.33, 129.84, and 149.94 (Ar-C), and 183.83 (>C=S). ^31^P-NMR (DMSO- d6, 162 MHz); δ ppm: 14.85 ppm. Elemental analysis calc. (%) for C_34_H_32_N_4_O_6_P_2_S_2_: C, 56.82; H, 4.49; N, 7.82; P, 8.62; S, 8.92; and O, 13.36; found: C, 54.97; H,4.36; N, 8.18; P, 8.83; and S, 7.91.

### 2.4. Synthesis of α-Thioureidophosphonate-Based Silver Nanoparticles

In 250 mL glass beaker, 0.6 g of each raw powder of MTP and BTP were dispersed individually in 150 mL of AgNO_3_ solution of 100, 500, and 1000 mg L^−1^ concentrations. Then, the prepared solutions were magnetically stirred (200 rpm) for 12 h, at ambient conditions. Afterward, the attained colloidal solutions were centrifuged, and the settled nanoparticles were washed with ethanol/deionized water and then oven-dried at 50 °C for further characterization [52].

### 2.5. Microorganisms and Media

Nutrient broth, Tryptic soy broth (TSB), and nutrient agar were purchased from Bacto, Australia. Mueller Hilton broth (Becton Dickinson, Sparks, MD, USA), DMSO and iodonitrotetrazolium chloride (INT) were procured from RandM marketing, Essex UK. The reference antibiotics ciprofloxacin, vancomycin hydrochloride, and ampicillin were supplied from Sigma Aldrich, Germany. Standard 96-wells microplate reader, pipette, 96-wells microtiter, 15 mL centrifuge tubes were obtained from Lab supply company, Cairo, Egypt. The crystal violet, glacial acetic acid, and 1×Phosphate-buffer saline were purchased from Thermo Fisher Scientific (Waltham, MA, USA). Clinical isolates of methicillin-resistant *Staphylococcus aureus* (*MRSA*), *Streptococcus mutans* (*S. mutans*), *Bacillus subtilis* (*B. subtilis*), *Pseudomonas aeruginosa* (*P. aeruginosa*), *Salmonella typhi* (*S. typhi*), and *Serratia marcescens* (*S. marcescens*) were acquired from National Research Center, Cairo, Egypt, along with their ATCC references (*MRSA*-ATCC 43300), (*S. mutans*-ATCC 35668), (*B. subtilis*-ATCC 6633), (*P. aeruginosa*-ATCC 27853), (*S. typhi*-ATCC 6539), and (*S. marcescens*-ATCC 13880), respectively.

### 2.6. Molecular Docking Study

Molecular docking studies were conducted using molecular operating environment (MOE) [53] with the aid of Discovery studio. Four proteins from different microorganisms of Gram-positive and Gram-negative bacteria (*MRSA*, *B. subtilis*, *P. aeruginosa*, and *S. typhi*) were downloaded for modeling study from the protein data bank (PDB codes: 4DKI, 2FQT, 5ZHN and 3ZQE), respectively [54,55,56,57]. Ligand and protein structural optimizations were applied by calculating partial charges, 3D protonation, strands correction, followed by energy minimization [58]. The selected docking protocol was induced fit, where the ligand active site was selected as a placement guide. Exclusion of pharmacophore annotations was selected. The gradient for energy minimization was 0.05, and MMFF94X was the force field by default [59].

### 2.7. Pharmacokinetics In Silico Screening

Pharmacokinetics prediction of the final compounds was performed in silico using the available software preADMET (https://preadmet.bmdrc.kr/) accessed on 20 April 2023.

## 3. Results and Discussion

### 3.1. Chemistry

#### 3.1.1. Synthesis and Structural Characterization of Thioureidophosphonate Compounds

**Scheme 1:**
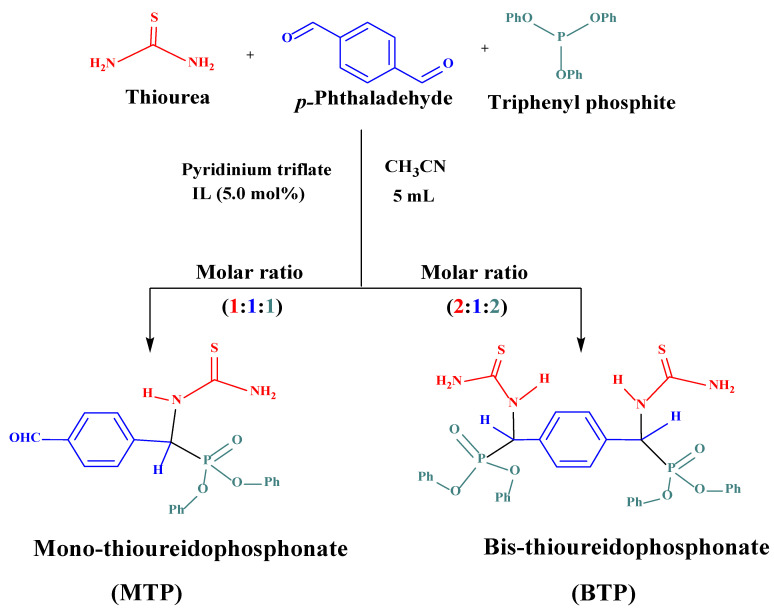
One pot-three component synthesis of MTP and BTP.

Two novel organophosphorus compounds were prepared in a facile one pot Kabachnik–Fields reaction via pyridinium triflate as, a green protic ionic liquid, as a Lewis acid catalyst [36]. Two different molar ratios of 1:1:1 and 2:1:2 were inserted, zeta-potential charge, for the three thiourea, terephthalaldehyde, and triphenyl phosphite reactants, giving mono and bis substituted thioureidophosphonates (MTP and BTP) (Scheme 1). Both reactions were performed in acetonitrile (CH_3_CN) as a solvent at room temperature with overnight stirring and followed by TLC using an eluent mixture of hexane–methylene chloride (3:1). Moreover, the attained yields reached around 91.72 and 87.71% for MTP and BTP, successively.

**Figure 1:**
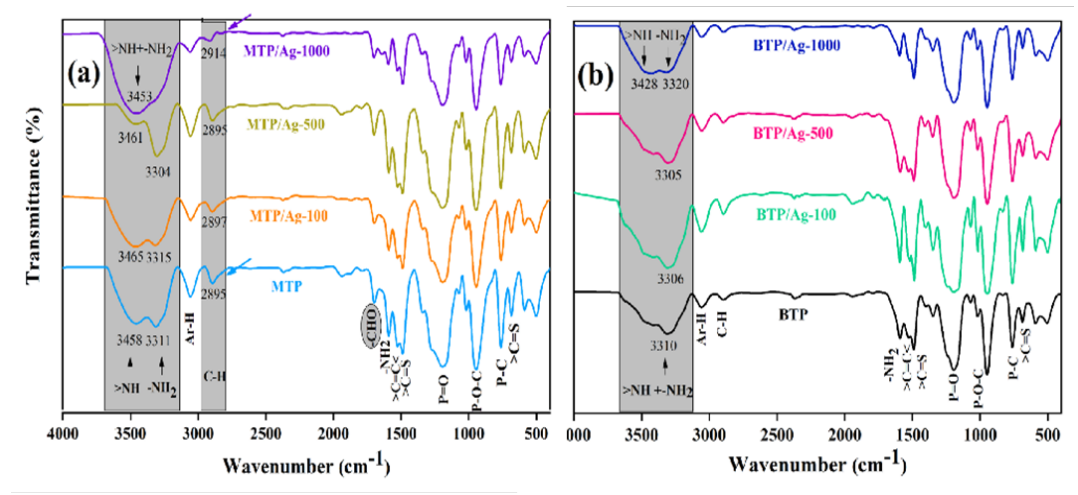
(**a**) FT-IR spectra of Ag NCs of MTP and (**b**) BTP compared to their AgNPs-free cores.

The chemical structures of TPs (MTP and BTP) were fully elucidated through FT-IR spectroscopy, (^1^H-, ^13^C-, and ^31^P) -NMR techniques, and elementary (CHNS) analysis, successively. Referring to the FT-IR spectra of raw MTP and BTP analogues (Figure 1), the absorption bands stretched at **ν^−^** = 3310/3458, 3058, and 1526 cm^−1^, indicated to primary/secondary amine (-NH_2_/>NH) and aromatic (C–H) and >C=C< bonds, consecutively. The aliphatic bands of C–H, -P=O, and P–C were recorded at **ν^−^** = 2896, 1196, and 762 cm^−1^, respectively. Moreover, two peaks were noticed at **ν^−^** = 945 and 948 cm^−1^, due to the presence of P–O–C bond in MTP and BTP, respectively. Remarkably, one distinctive vibration band was observed, indicating the stretching of -CHO in MTP compound. Apart from this band observed at **ν^−^** = 1698 cm^−1^, the bending vibration of (>NH) was assigned at **ν^−^** = 1591 cm^−1^. Furthermore, symmetrical and asymmetrical vibrations of (>C=S) were viewed at **ν^−^** = 1490 cm^−1^ and **ν^−^** = 686–687 cm^−1^ for both characterized compounds, successively. Thus, the FT-IR spectroscopic tool reflected the complete formation of raw TP products.

The **^1^**H-NMR spectra were performed in deuterated DMSO, elucidating the formation of both TPs with notably characteristic chemical shifts (δ) values in the aliphatic and aromatic regions. First, the chiral proton of P–C–H appeared at δ = 5.50 ppm (1H, d), while all Ar–H were demonstrated as combined peaks in δ range of 6.63–7.99 ppm (Ar-H, m, 14H). Likewise, the 2^ry^ (>NH) proton was depicted as a broad singlet at δ = 9.76 ppm (br.s, 1H), while the 1^ry^ (-NH_2_) protons were observed as a broad doublet at δ = 9.40–9.52 ppm (br, d, 2H). The singlet peak at δ = 10.04 ppm (s, 1H) illustrated the presence of a freely unreacted formyl (-CHO) group, affirming the successful synthesis of MTP (Figure S1a). On the other hand, two chiral centers of P–C–H with a singlet peak were disclosed at δ = 5.93 ppm (s, 2H) in BTP. Further, protons of the five aromatic rings were uncovered as multiplet peaks with lower δ = 6.54–7.61 ppm (m, 24H) in comparison to MTP. Similarly, the two terminal amine groups were noted as a broad multiplet at δ = 9.21–9.33 ppm (br. m, 4H). Lastly, the two secondary amino groups (>NH) were illustrated as a broad singlet peak at δ = 9.38 ppm (Figure S1b). Moreover, ^13^C-NMR spectra of both TPs conducted in deuterated DMSO. The MTP spectrum gave representative peaks of P–C–H chiral carbon displayed at δ = 54.59 and 56.16 ppm while the aromatic C–Hs were observed at δ = 115.24, 120.22, 120.53, 125.39, 127.96, 128.92, 129.90, and 149.80 ppm. In addition, thiocarbonyl (>C=S) and formyl (-CHO) carbon were shown at δ = 183.84 and 192.73 ppm, sequentially (Figure S1c). Two significant signals of chiral centers in BTP were identified at δ = 54.50 and 56.06 ppm. Furthermore, the aromatic carbon atoms were clarified in eight signals with δ = 115.26, 120.29, 120.86, 125.40, 127.50, 129.33, 129.84, 149.94 ppm, while (>C=S) carbon was noticed at δ = 183.83 ppm (Figure S1d). The phosphorus atoms in MTP and BTP were individually resonated as a single peak at δ = 14.08 and 14.85 ppm, respectively (Figure S1e,f). Additionally, the elemental analysis of the as-prepared TPs manifested the successful reactions between thiourea, terephthalaldehyde, and triphenyl phosphite, individually (Table S1) (Figure S2).

**Scheme 2:**
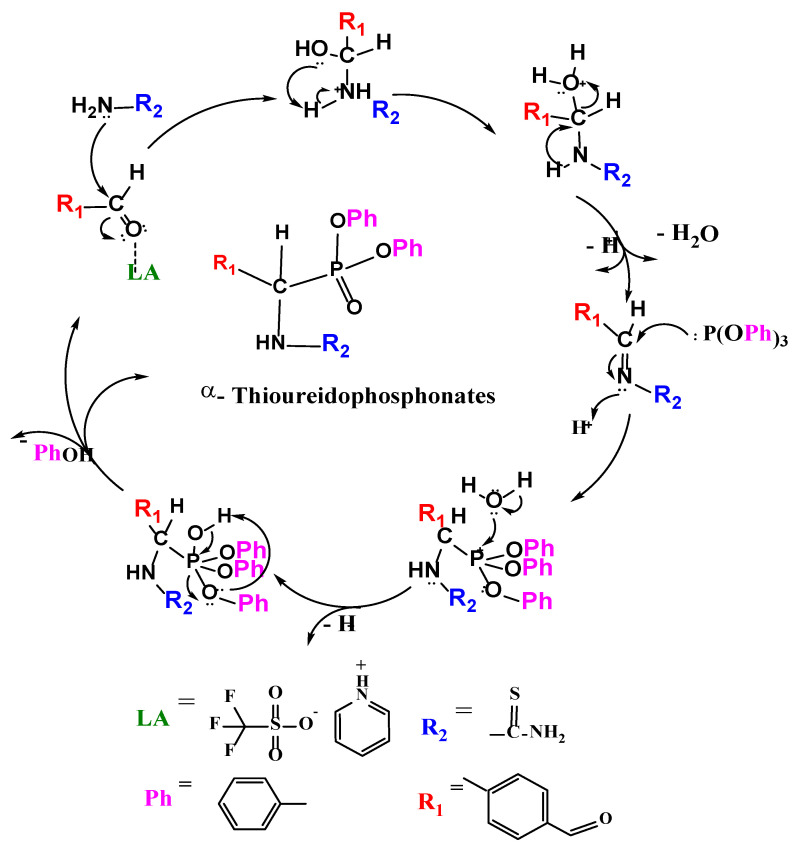
The proposed mechanism of the one-pot three-component reaction catalyzed by pyridinium triflate as protic ionic liquid.

Accordingly, the mechanistic route of the reaction occurred through two major steps: (a) the in situ generation of Schiff base via activation of the formyl group by Lewis acid (LA) catalyst; this facilitated the condensation reaction between thiourea and terephthalaldehyde via nucleophilic addition of a (thiourea) nitrogen lone pair to the electrophilic carbon of the activated carbonyl group of -CHO; (b) the nucleophilic attack of the (triphenyl phosphite) phosphorus atom on the electrophilic carbon of the imine moiety (>C=N-), followed by the extrusion of the phenol molecule through formation of the in situ phosphonium salt and hydroxy phosphite intermediates (Scheme 2) [36].

#### 3.1.2. Fabrication and Structural Elucidation of Thioureidophosphonates Capped Silver Nanoparticles

Well dispersed AgNPs were produced via one-pot synthesis method using thioureidophosphonates (TPs) as dual-functioning reducing and capping agents. This affords spherical AgNPs different dispersion patterns based on the reaction conditions of AgNPs precursor concentrations and the structure of TPs (Figure S3).

The structure and dispersion of AgNPs in the developed nanoscale formulations were fully elucidated using different tools. The FT-IR spectra of MTP incorporated with different molar ratios of silver loading (100, 500, and 1000 mg Ag L^−1^) illustrated meaningful changes in the characteristic absorption bands of capping agent (MTP) (Figure 1a). Notably, the position of the secondary amine (>NH) of MTP at ν = 3458 cm^−1^ was shifted to ν = 3465, 3461, and 3453 cm^−1^ as for 100, 500, and 1000 mg Ag L^−1^, respectively. Furthermore, another successive shift occurred from ν = 3311 cm^−1^ to 3315, 3304, and 3453 cm^−1^, revealing that terminal amine (-NH_2_) was also involved in the interaction and reduction of Ag^+^ ions in all concentrations of 100, 500, and 1000 mg Ag L^−1^, respectively. Further, the aliphatic (H-C-P) bond was dramatically shifted from ν = 2895 cm^−1^ to ν = 2914 cm^−1^ in MTP/Ag-1000. On the other side, the FT-IR spectra of BTP and its (100, 500, and 1000) AgNP dispersions displayed similar changes, with a substantial shifting of amine functional groups from ν = 3310 cm^−1^ to ν = 3306, 3305, and (3320–3428) cm^−1^, consecutively (Figure 1b). Hence, FT-IR technique validated the real contribution of raw TPs in the reduction and capping process and, in turn, the formation of AgNPs (Table S2). Basically, a gradual change in the color of Ag NC solutions was noticed when concentration of dispersed AgNO_3_ was altered from 100 to 500, and then 1000 mg Ag L^−1^ (Figure S4).

**Figure 2:**
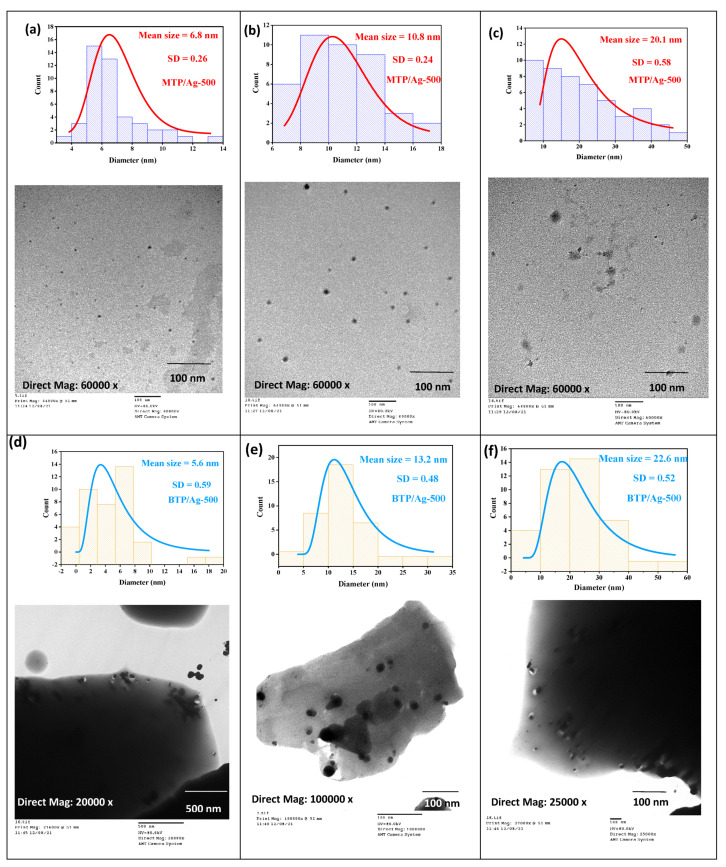
TEM images and size distributions by the histogram in (**a**–**c**) for MTP/Ag-500 NC with 100 nm scale bar, and in (**d**–**f**) for BTP/Ag-500 NC with 500 nm scale bar (**d**) and 100 nm (**e**,**f**), respectively.

The morphology and dispersion nature of developed silver nanoparticles (AgNPs) were scrutinized using microscopic techniques. Accordingly, the TEM imaging for MTP/Ag-500 displayed good dispersion of spherical AgNPs with an average mean size range of 6.8–20.1 nm (Figure 2a–c). On the other hand, some irregularly spherical and agglomerated AgNPs were observed for BTP/Ag-500 with a mean size range of 5.6–22.6 nm. Remarkably, the non-agglomerated AgNPs on MTP could be ascribed to the smooth morphological structure of MTP facilitating the surface interactions with Ag^+^ ions, while the higher density of chelating centers in BTP significantly improved the reduction rate of Ag^+^ ions (with deeper color); thus, some smaller AgNPs were attained with the BTP surface (Figure 2d–f). The emergence of aggregated AgNPs could be related to the steric hinderance of BTP structure lowering the capping efficiency [60].

**Figure 3:**
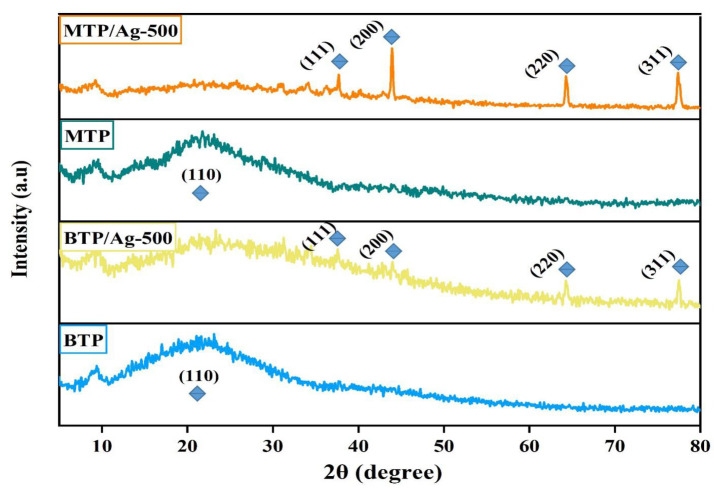
XRD patterns of MTP, MTP/Ag-500, BTP, and BTP/Ag-500 structures.

Moreover, the crystallinity of the as-synthesized TPs after AgNPs functionalization was investigated compared to that of raw capping agents using XRD analysis. First, broad XRD patterns were individually centered at 2 theta (2θ) = 21.7° with (110) diffraction phases, reflecting the amorphous structure of both raw MTP and BTP analogues. The emergence of broad patterns stemmed from the strong inter- and intra-molecular hydrogen bonding in raw TP cores [36]. Further, well resolved and sharper diffraction peaks were noticed for both MTP and BTP/Ag-500 at 2θ angles of 37.97°, 43.83°, 64.16°, and 77.49° corresponding to 111, 200, 220, and 311 Bragg peaks of metallic silver in a face-centered cubic (FCC) structure, progressively (Figure 3). Consequently, these results were in agreement with the unit cell structure of silver lines of JCPDS (File No. 04–0783) [61].

**Figure 4:**
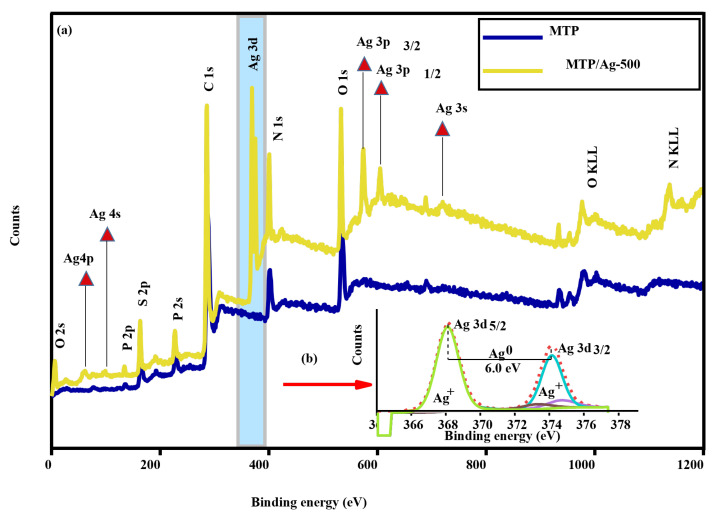
(**a**) XPS spectra of the raw MTP and MTP/Ag-500 nanocomposite and (**b**) the inset figure of Ag3d peak deconvoluted into Ag3d _5/2_ and _3/2_ for MTP/Ag-500.

To validate the most significant changes that occurred to raw functional groups after Ag^+^ ions incorporation and formation of AgNPs, X-ray photoelectron spectroscopy analysis (XPS) was carried out for MTP/Ag-500. The XPS results of MTP/Ag-500 were studied based on evaluating the changes in binding energies (BEs), atomic percentages (%), electronic properties, and chemical composition of MTP before and after Ag^+^ ion interactions. Five elemental signals of C 1s (at BE: 285.84–287.23 eV), N 1s (400.72–401.63 eV), O 1s (533.02–534.81 eV), P 2p (134.32–134.57 eV), and S 2p (163.19–164.19 eV) were mainly evolved, consecutively (Figure 4a and Figure S5). Regarding the spectrum of MTP/Ag-500 composite, two intensive peaks appeared at BEs of 368.11 and 374.14 eV were ascribed to Ag 3d_5/2_ and Ag 3d_3/2_ of Ag^0^ particles, respectively. In addition, the inset manifested that Ag 3d_5/2_ peak was deconvoluted into two peaks observed at 368.02 eV and 368.84 eV, while Ag 3d_3/2_ was also divided into two peaks of 374.0 eV and 374.73 eV, respectively (Figure 4b) [62]. However, some other peaks successively emerged at BEs of 366.27, 369.03, and 373.30 eV. They could have originated from the incomplete reduction of Ag^+^ ions in the form of Ag^+^-N or Ag^+^-O. On top of this, the rough calculation results evaluated that around 92.28 atomic % of Ag^+^ ions were reduced to Ag^0^, proving the prominent ability of MTP functional groups in the interaction and reduction of Ag^+^ ions to Ag^0^ particles. Moreover, a series of Ag 3s, Ag 3p_1/2_, Ag 3p_3/2_, Ag 4s, and Ag 4p signals appeared at BEs of 719, 604, 573, 98, and 60 eV, respectively. Thus, the XPS spectrum of the MTP surface exposed to Ag^+^ ions was altered to validate the changes in binding and chelation routes. Furthermore, some changes were primarily observed via minor shifts of BEs from (0.04–0.46) eV, besides considerable changes in the atomic percentages (%) of raw elemental functional groups that were estimated from 0.72–25.57%. Basically, these notably affected functional groups were ordered, respectively, in a descending mode according to changes in BEs as follows: C–S (2p_1/2_) > C–C, C–H, and C=C > HC=O and -OH (H_2_O) > C–S (2p_3/2_) > P–C and P–O (2p_1/2_) > >NH and -NH_2_ > C=S > C–N > π–π* sat, benzene rings, and C=S > C-OH, P–O, and C–O–C > C–O, C–N, C–P, and C=O (Table S3). Furthermore, after Ag^+^ ions complexation and formation of additional O–H (from H_2_O) on the MTP, the main signal deconvolution assessed that Ag^+^ ions may be sorbed in their hydrated form (i.e., their solvated form). In this regard, the changes that occurred in BEs after AgNPs decoration were consistent with the results discussed before.

### 3.2. Antibacterial Activity

The antibacterial potency of synthesized thioureidophosphonates (TPs) and their fabricated AgNPs-based composites was evaluated against several strains. The antibacterial activity using the agar well diffusion method was conducted against six clinical bacterial strains and their ATCC references of Gram-positive (methicillin-resistant *Staphylococcus aureus* (*MRSA*), *Streptococcus mutans*, and *Bacillus subtilis*) and Gram-negative (*Pseudomonas aeruginosa*, *Salmonella typhi*, and *Serratia marcescens*) bacteria as well. Moreover, zone of inhibition diameters, minimum inhibitory concentration (MIC), minimum bactericidal concentration (MBC), time–kill kinetics, antiadhesion activity, and antibiofilm assay with morphological investigation were assessed for all developed compounds.

#### 3.2.1. Bactericidal Properties

##### The Killing Action against Gram-Positive Strains

**Figure 5:**
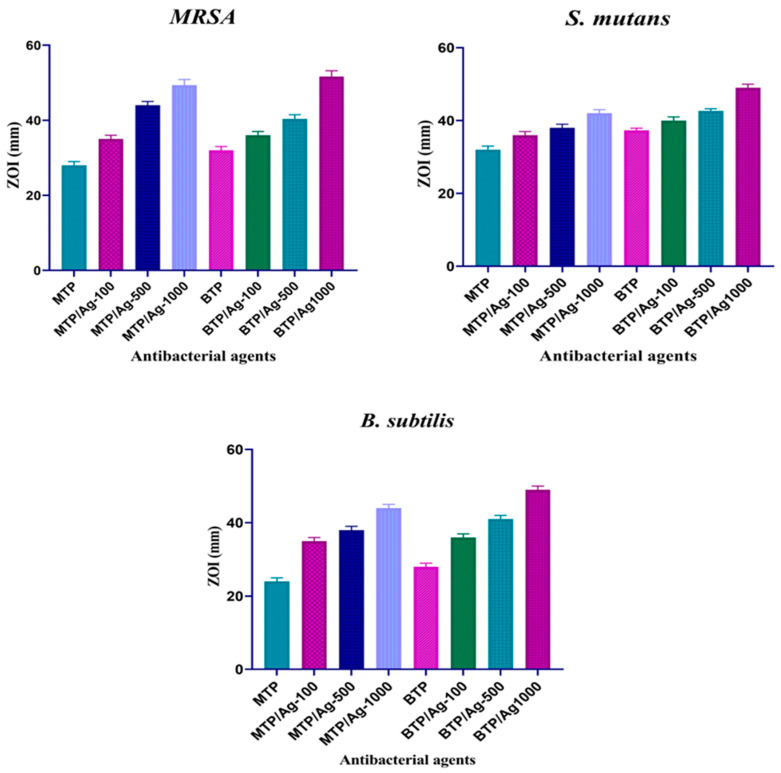
ZOI values of all antibacterial agents towards Gram-positive bacterial strains. The experiments were performed in triplicates.

MTP and BTP showed significant resistance against methicillin-resistant *Staphylococcus aureus* (*MRSA*), *Streptococcus mutans* (*S. mutans*), and *Bacillus subtilis* (*B. subtilis*), compared to Ampicillin and Ciprofloxacin as reference antibiotics (Table 1, Table 2 and Table 3). First, MTP exhibited higher MIC (0.625 mg/mL) than BTP against *MRSA*, reducing MIC values by 5 and 1.2 times compared to Ampicillin and Ciprofloxacin controls, respectively. On the other hand, MIC attained for BTP was equal to 0.3125 mg/mL, which had twofold better antibacterial superiority than MTP against *MRSA*. The antibacterial evaluation was performed against *S. mutans* with respective MIC values of 0.3125 and 0.156 mg/mL for MTP and BTP, while the estimated MIC values towards *B. subtilis* reached 0.3125 and 0.0781 mg/mL. Compared to Ampicillin and Ciprofloxacin, MTP reduced MIC values by 5 times towards *S. mutans*, while the relative decline in MIC reached 12.8 and 5 times against *B. subtilis*, consecutively. BTP had more structural diversity, allowing it to be more powerful than MTP, minimizing MIC values by two and four times against *S. mutans* and *B. subtills*, sequentially. Screening both MTP and BTP against *MRSA* (ATCC-43300) resulted in higher MIC values = 1.25 mg/mL for both analogues. MTP and BTP recorded MICs at lower concentrations of 0.625 and 0.3125 mg/mL against *S. mutans* (ATCC-35668) and *B. subtilis* (ATCC-6633), progressively. The zone of inhibition (ZOI) was measured for each strain with DMSO as a negative control. Including the diameter of each well (6 mm) before treatment, MTP gave ZOIs in a range of 28 ± 1.0, 30 ± 1.00, and 24 ± 1.00 mm, whereas the treatment with BTP increased the ZOIs width towards *MRSA*, *S. mutans*, and *B. subtilis* strains, respectively. Both TPs with MBC/MIC ratio of 2(+) have bactericidal properties, unlike Ampicillin and Ciprofloxacin. TPs loaded with silver nanoparticles (AgNPs) (100, 500, and 1000 mg Ag L^−^^1^) were used to investigate the antibacterial properties towards three strains accompanied with a dose-dependent improvement in susceptibility. A clear MIC decline to very minute concentrations was appreciated from 0.156 to 0.1525 × 10^−3^ mg/mL with the three strains. The MIC of MTP significantly dropped by (4 × MIC) after treating *MRSA* and *S. mutans* with MTP/Ag-100. Afterward, MTP/Ag-500 and MTP/Ag-1000 showed increased bactericidal properties against both strains, i.e., 16 and 2049 times sequentially. The treatment of MTP/Ag-(100, 500, and 1000) to *B. subtilis* increased the potential bactericidal activity by 16, 128, and 1024 times, respectively. BTP/Ag-100 showed a more significant decline in MIC values by two and eight times against *MRSA* and *B. subtilis*, compared to MTP/Ag NCs. BTP/Ag-500 had less biocidal effects by 4 and 16 times that of MTP/Ag-500 on *MRSA* and *B. subtilis*, consecutively. This reflected that BTP and AgNPs have less synergistic interactions than MTP. However, BTP/AgNPs had the same bactericidal activity as MTP/AgNPs against *S. mutans* in 100 and 500 dispersions. Noteworthy, MTP and BTP loaded with 1000 mg L^−^^1^ resulted in a decrease in MICs by 2049 times against *MRSA* while BTP/Ag-1000 showed higher MIC against *S. mutans* and *B. subtilis* by only 1023 and 512 times, respectively (Table 1, Table 2 and Table 3). Further, ZOI diameters increased towards *MRSA* treated by TP/Ag NCs in all concentrations of 100, 500, and 1000 mg Ag L^−1^. After AgNPs incorporation, increased ZOI diameters from 28 ± 1.00 to 35 ± 1.00, 44 ± 1.00, and 49 ± 1.50 mm were achieved for MTP with 100, 500, and 1000 mg Ag L^−^^1^, successively (Figure S6). Similarly, ZOI values were promoted from 32 ± 0.60 to 36 ± 1.00, 40 ± 0.60, and 52 ± 1.00 mm after treatment with 100, 500, and 1000 BTP/Ag NCs, progressively. Moreover, MTP improved ZOI values against *S. mutans* from 30 ± 1.00 to 36 ± 1.00 (MTP/Ag-100), 38 ± 1.00 (MTP/Ag-500), and 42 ± 1.00 mm for MTP/Ag-1000, respectively. Likewise, ZOIs exhibited a gradual increase from 37 ± 1.00 in BTP to 40 ± 1.00 (BTP/Ag-100), 43 ± 0.60 (BTP/Ag-500), and 49 ± 0.100 mm (BTP/Ag-1000). MTP and BTP gave higher ZOIs with diameters of 35 ± 1.00, 38 ± 1.00, and 40 ± 1.00 mm and 36 ± 1.00, 41 ± 1.00, and 41 ± 1.00 mm, respectively, for *B. subtilis* (Figure 5, Table 1, Table 2 and Table 3, and Figures S6, S8 and S9). Briefly, MTP/Ag NCs showed dose-effective antibacterial activity against all studied Gram-positive bacterial strains.

##### The Killing Action against Gram-Negative Strains

**Figure 6:**
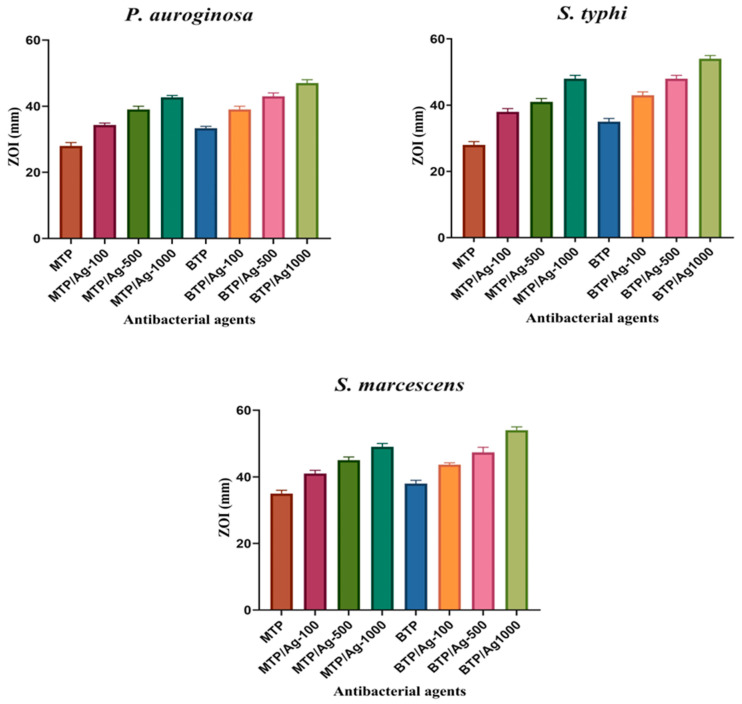
ZOI values of all antibacterial agents towards Gram-negative bacterial strains. The experiments were performed in triplicates.

Contrarily, TP analogues had more selective antibacterial properties towards *Pseudomonas aeruginosa* (*P. aeruginosa*), *Salmonella typhi* (*S. typhi*), and *Serratia marcescens* (*S. marcescens*) than previously screened Gram-positive bacteria and reference antibiotics (Figure S13). Towards *P. aeruginosa*, MTP showed a similar MIC value to MRSA at 0.625 mg/mL, which was reduced by 10 and 2.5 times with Ampicillin and Ciprofloxacin references. BTP had an 8-fold lower MIC (=0.078 mg/mL) against *P. aeruginosa* than MTP, indicating higher susceptibility towards Gram-negative strains. Both MTP and BTP compounds had excellent inhibitory properties against *S. typhi* and *S. marcescens*, as their MIC ratio was reduced fourfold. Regarding the Ampicillin and Ciprofloxacin, 5× and 20× MIC reductions by MTP were recorded against *S. typhi*, while 5.0 and 1.2× MIC were dropped with *S. marcescens*. On the contrary, BTP decreased the MICs of Ampicillin and Ciprofloxacin by 20 and 80 times towards *S. typhi*, and to 20 and 5 times against *S. marcescens*, respectively. Furthermore, the selectivity of these TP derivatives was determined against *P. aeruginosa* (ATCC-27853), *S. typhi* (ATCC-6539), and *S. marcescens* (ATCC-13880). Meanwhile, the MICs obtained by inoculating both ATCC isolates of *P. aeruginosa* and *S. marcescens* with MTP were 1.25 mg/mL for *S. typhi* (ATCC-6539). The selectivity of MTP was more effective, affording an MIC value of 0.625 mg/mL. BTP had two lowered MIC values against *P. aeruginosa* (ATCC-27853), *S. typhi* (ATCC-6539), and *S. marcescens*, with 0.3125 mg/mL for *P. aeruginosa* and 0.625 mg/mL for *S. marcescens* (ATCC-13880). On the other hand, ZOI results of MTP and BTP against *P. aeruginosa, S. typhi*, and *S. marcescens* were 28 ± 1.00, 28 ± 1.00, and 35 ± 1.00 mm for MTP, while 33 ± 0.60, 35 ± 1.00, and 38 ± 1.00 mm for BTP, consecutively. Therefore, MBC values ranged from 1.25 to 0.156 mg/mL, resulting in a significant bactericidal efficacy against all bacterial strains with MBC/MIC ratio = 2(+). Notably, AgNPs improved the antibacterial susceptibility of TP cores, lowering MIC values from 0.01953 mg/mL to 7.629 × 10^−^^5^ mg/mL. Basically, MTP/Ag-100 minimized the MIC concentration of raw MTP by around 32 times towards *P. aeruginosa,* and 16 times for both *S. typhi* and *S. marcescens*, successively. MTP/Ag-500 reduced MICs by 256 times for *P. aeruginosa* and 128 times for *S. typhi* and *S. marcescens* compared to raw MTP. For MTP/Ag-1000, MTP displayed an elevated antibacterial activity towards *P. aeruginosa*, *S. typhi*, and *S. marcescens*. Nevertheless, the net antibacterial activity of BTP was higher than MTP, and the combinatorial interactions of AgNPs with MTP were more tunable, unlike BTP. In the meantime, BTP/Ag-100 lessened the MIC ratio to raw BTP by fourfold towards *P. aeruginosa* and eightfold against both *S. typhi* and *S. marcescens.* BTP/Ag-500 had a remarkable bactericidal effect on *S. typhi* up to 128 times. Then, the BTP/Ag-1000 reduced the dose of MIC to 512 times with *P. aeruginosa* and 1023 times with both *S. typhi* and *S. marcescens*, respectively. Pertinently, BTP loaded with AgNPs manifested less powerful bactericidal potency than MTP capped AgNPs (Tables S10–S12). Further, wider ZOI diameters for Gram-negative strains were recorded over Gram-positive strains. In case of MTP after AgNPs dispersion, the ZOIs were increased from 28 ± 1.00 to 34 ± 0.60, 39 ± 1.00, and 43 ± 0.60 mm, whilst BTP/Ag NCs enhanced the gradual enlargement of raw diameter from 33 ± 0.60 (BTP) to 39 ± 1.00, 43 ± 1.00, and 47 ± 1.00 mm in the studied ranges (i.e., 100, 500, and 1000), consecutively. In a dose-dependent manner, *S. typhi* inoculated with MTP/Ag NCs yielded ZOI values of 38 ± 1.00, 41 ± 1.00, and 48 ± 1.00 and 43 ± 1.00, 48 ± 1.00, and 54 ± 1.00 mm for BTP/Ag NCs, sequentially. AgNPs anchoring increased the ZOIs of raw MTP and BTP against *S. marcescens* from 35 ± 1.00 to 41, 45, and 49 ± 1.00 and 38 ± 1.00 to 44, 47, and 54 ± 1.00 mm, consecutively (Figure 6, Table 4, Table 5 and Table 6, and Figures S10–S12. In summary, the effective synergy between TPs and AgNPs displayed great results with all studied bacterial strains. The presence of TP moiety as a self-biologically active material was effective in comparison to other reported AgNP-based antibacterial agents (Table S4).

#### 3.2.2. Time–Kill Assay

**Figure 7:**
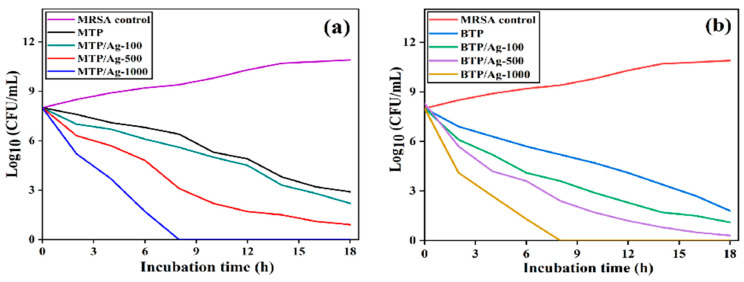
Bactericidal kinetics of (**a**) MTP, and MTP/Ag NCs; and (**b**) BTP, and BTP/Ag NCs against *MRSA*. Untreated *MRSA* cells were employed as a growth control (The experiments were performed with three duplicates).

This biological test was executed to explore the bactericidal activity of TP derivatives and their AgNPs-based composites towards bacterial growth over a period [63]. When bacterial cells at a concentration of 1 × 10^8^ CFU/mL were incubated with all raw and AgNPs formulations, the *MRSA* growth rate decreased with time compared to the incremental growth of untreated *MRSA*. Interestingly, the viable cells count (CFU/mL) started to dramatically decline after 1 h of treatment (Figure 7). In the meantime, both raw MTP and BTP compounds reduced the number of surviving cells to (2.9 log_10_) and (1.8 log_10_) CFU/mL after 18 h of incubation, respectively. However, once *MRSA* was treated with TP/Ag NCs, the picture was changed where the MTP/Ag-100 induced the killing potential with cell count reductions to (2.2 log_10_) and (0.9 log_10_) CFU/mL for MTP/Ag-500, consecutively. More significant reductions of the cultural population of *MRSA* were achieved to (1.1 log_10_) and (0.3 log_10_) CFU/mL for BTP/Ag-100 and BTP/Ag-500, progressively. Despite the higher killing efficacy of BTP/Ag NCs than MTP/Ag NCs, the combination between AgNPs and MTP brought more bactericidal advantages over BTP, which was observed from the gradual improvement of killing (%) between dose 100 and 500 for both AgNPs formulations. Remarkably, the entire population was reduced and killed within 8 h, for both MTP (BTP)/Ag-1000 composites. Interestingly, the bactericidal rates towards *MRSA* were previously confirmed by both TP/Ag-1000; where both reduced MBC values to the same degree of 2049 times (Table 1). Based on several reported studies, it was interesting to mention that the rapid bactericidal ability of raw TPs (as aminophophonate-based derivatives), could be attributed to their ability to (1) deactivate the potential of cytoplasmic membrane and interfere with DNA nitrogenous bases through aromatic pi-pi bonds. Besides, (2) to generate reactive nitrogen/oxygen species (RNS/ROS) that caused peroxidation of lipid, glutathione (GSH) conversion to glutathione disulfide (GSSG), and thereby cell malfunction followed by death of bacteria, respectively [64,65]. Additionally, AgNPs exhibited a more considerable bactericidal activity which may be due to their toxic effects on bacterial cell wall and cytoplasmic membrane components, resulting in the reduction of cell wall integrity, functionality, defense system, and metabolic activity [18,66]. Thus, the time-dependent kill study revealed that the investigated compounds were time- and dose-effective bactericidal agents.

#### 3.2.3. Antiadhesion Activity

**Figure 8:**
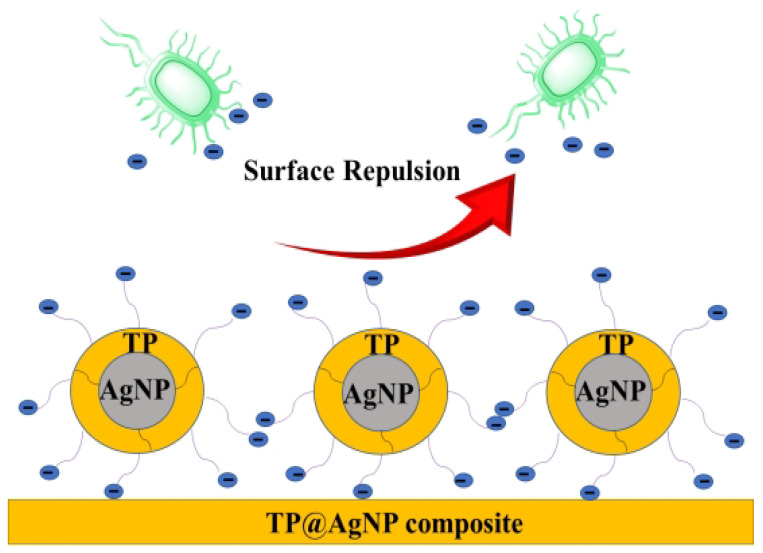
Schematic representation of the surface repulsion of the negatively charged TP/Ag NCs with *MRSA* (ATCC-43300) surface.

**Figure 9:**
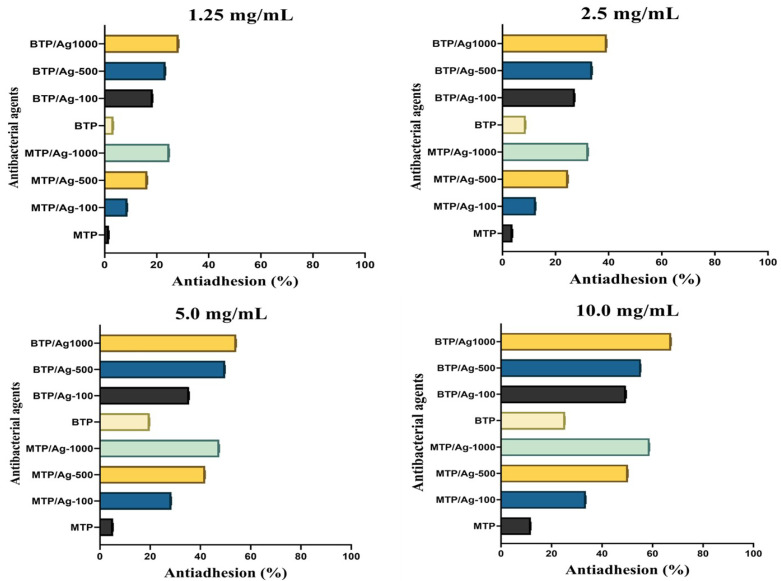
Antiadhesion assay of tested compounds towards *MRSA* (ATCC-43300) based on quadruplicate results with standard deviation, (n = 4).

Bacterial adhesion to any object surface is an initial precondition for bacterial growth through biofilm and infection processes. Additionally, there are some crucial parameters controlling the surface tendency for adhesion; these parameters include hydrophobicity, surface energy, and zeta potential charges [67,68]. Hence, fabricating and engineering of novel antiadhesive surfaces with repelling properties against bacteria has become a vital step for investigation (Figure 8). In the current study, all prepared compounds were incubated with high concentration (1 × 10^6^ CFU/mL) of *MRSA* (ATCC-43300) suspensions for 18–24 h, using vancomycin hydrochloride (VAN) as a positive control, and the negative control was tryptic soy broth (TSB). Antiadhesion activity was quantitatively estimated against *MRSA* (ATCC-43300) on the microplate reader at λ_max_ = 570 nm, validating an increase in *MRSA* (ATCC-43300) detachment in a dose-dependent manner within 1.25, 2.5, 5.0, and 10 mg/mL (Figure 9 and Table 7). It was demonstrated that the degree of bacterial adhesion decreased with the increasing hydrophobicity of substrates due to the hydrophilic properties of most strains, such as Gram-positive bacteria. Moreover, there is a direct relation between surface energy and the hydrophobic structure of adhesive surfaces. It has been reported that the higher surface energy stems from the substrates terminated with polar functional groups, such as -NH_2_, while those composed of hydrophobic carbon moieties may minimize the surface energy [68]. In conclusion, a higher hydrophobic structure associated with inferior surface energy affords good repelling properties towards bacteria. Accordingly, BTP exhibited higher antiadhesion activity in all utilized concentrations towards *MRSA* (ATCC-43300) than MTP, which could be attributed to its superior hydrophobic characteristics as the non-polar parts (aromatic rings) exceeded the polar ones, based on its molecular formula (M.F), namely, C_34_H_32_N_4_O_6_P_2_S_2_, comparable to MTP with M.F of C_21_H_19_N_2_O_4_PS. Moreover, the electrostatic repulsion forces assessed between the negatively charged MTP/Ag NCs and *MRSA* (ATCC-43300) surface played a critical role [69]. As a result, MTP capped AgNPs in 100, 500, and 1000 formulations ascertained more significant increases (relative to raw) in the detachment ability against *MRSA* (ATCC-43300), most notably for 5 and 10 mg/mL. This may originate from the repelling affinity of the negatively charged Ag NCs, e.g., MTP/Ag-1000 with zeta potential = −27.5, mv towards *MRSA* (ATCC-43300). In contrast, inferior repelling rates were estimated for BTP-based AgNPs, especially at 5 and 10 mg/mL, which could be attributed to the tendency of BTP/Ag NCs to form more constrained molecules at high concentrations, thus lowering the contact surface area for repulsion, in addition to their less negative zeta charge like that of BTP/Ag-1000 in −12.4 mv in comparison to that of MTP/Ag-1000 (Figure S14) [70]. Additionally, at 1.25 and 2.5 mg/mL, BTP/Ag NCs displayed more notable repellent properties than that of MTP/Ag NCs at the same concentration. Thus, a greater contact surface area of BTP/Ag NCs could be obtained at lower concentrations, providing more hydrophobic and repelling characteristics. Further, the positive control (VAN) used at (100 mg/mL) showed lower antiadhesion activity (8.9%) compared to TSB as a negative control. The hydrophilic and polycationic characteristics of VAN towards the *MRSA* (ATCC-43300) surface may relate to this weak detachment activity [71]. On this account, the TP/Ag NCs proved to be efficient antifouling surfaces against multidrug-resistant Gram-positive bacteria such as *MRSA* (ATCC-43300).

#### 3.2.4. Antibiofilm Assay

**Figure 10:**
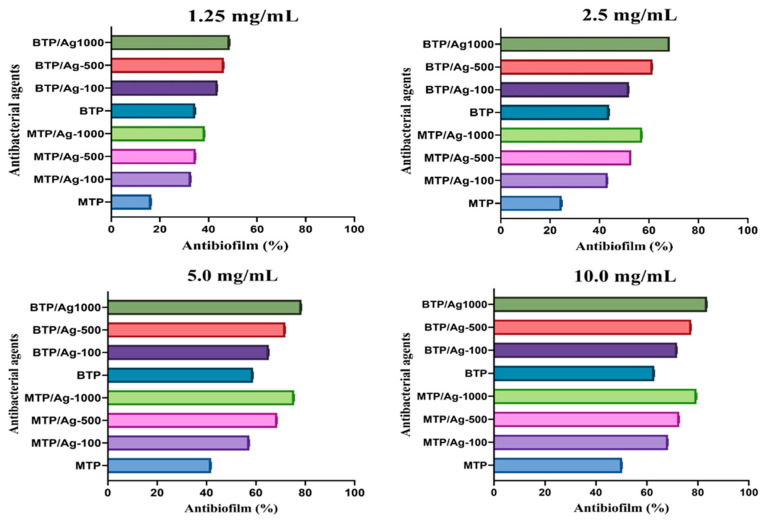
Biofilm eradication ability of tested compounds towards *MRSA* (ATCC-43300) based on quadruplicate results with standard deviation, (n = 4).

Methicillin-resistant *Staphylococcus aureus* (*MRSA*) is one of the most opportunistic and resistant pathogens, being able to produce an extracellular polymeric substance (EPS) inducing biofilm growth prior to the occurrence of cross infection [72]. The antibiofilm activity of the prepared compounds was tested against *MRSA* (ATCC-43300) as a Gram-positive bacterium with reference to vancomycin hydrochloride (VAN) as a positive control. The biofilm disintegration activity (%) was validated by the microtiter plate assay using a semi-quantitative analysis based on crystal violet staining. Regarding 96-well plates treated with raw MTP and BTP, the biofilm (violet color circle) was slightly eliminated, while the gradual removal was notably viewed by six TP/Ag NCs, (Figures S15 and S16). Therefore, these results suggested the superb antibiofilm potency of TP-wrapped AgNPs composites. Moreover, the decline (%) of biofilm formation was determined through a quantitative analysis appreciated by the photometric microplate reader at λ _max_ ≈ 570 nm with respect to the negative control of tryptic soy broth (TSB). The results showed a significant reduction in biofilm growth when treated with raw TPs, even after 24 h of incubation. The biofilm inhibition (%) was promoted with the gradual increase in the concentrations of tested compounds in the 1.25, 2.5, 5.0, and 10.0 mg/mL (Table 8, Figure 10). Yet, from the economic point of view, the 5.0 mg/mL concentration for all incubated formulations was thought to be the most synergistic and effective dose. The anti-biofilm activity of the positive control (VAN) reached 43 ± 0.17% when used even at 100 mg/mL, while raw BTP demonstrated higher (58.8%) antibiofilm activity than MTP (41.9%) at 5.0 mg/mL, which could be due to higher number of bio-active groups in BTP. Thus, the antibiofilm assay showed the strong potential of TP conjugates to disrupt biofilm EPS matrices of *MRSA* (ATCC-43300). The studied TP cores (α-aminophosphonate derivatives) are considered structural bio-isosteres of α-amino acids (peptides) with a common amine functional group, but the carboxylic groups in α-amino acids are replaced with related phosphorous-containing moieties such as phosphonic acid. However, the reported data about their antibiofilm mechanism of action towards MRSA biofilm are directed for hybrid α-APs; thus, the inhibitory actions of mentioned α-APs analogues were proposed [65,73]. First, the interference with the quorum sensing (QS) system, which represents a major source of biofilm nutrition and protection, could be a reason [74]. Moreover, the interaction with the extracellular DNA (e-DNA) of biofilm affects its integrity and biological functionality. The generation of reactive oxygen/nitrogen species (ROS/RNS) could cause oxidative stress as reported for based α-AP derivatives [65]. It was assessed that phosphorous-containing groups such as phosphonate compounds tend to inhibit cell wall synthetic enzymes and DNA bases and increase the permeability of bacterial cell membrane [75,76].

After AgNPs functionalization, biofilm inhibition capability (%) was promoted for both MTP and BTP in all studied concentrations (1.25, 2.5, 5.0, and 10.0 mg/mL), consecutively. The most effective and synergistic concentration needed for the disruption of MRSA (ATCC-43300) biofilm was observed at 5.0 mg/mL for both TP/Ag NCs. For 10.0 mg/mL, the biofilm eradicating ability for both MTP(BTP)/Ag-1000 reached maximum values at 79.5 and 83.6%, progressively (Table 8, Figure 10). Nonetheless, higher antibiofilm rates were accomplished for BTP/Ag NCs than that of MTP, the antibacterial effect of AgNPs showed a promising synergy with raw MTP conjugates over that of BTP, exhibiting improved toxicity when compared to TPs alone. Meanwhile, many reports showed that AgNPs affect biofilm integrity; smaller and spherical metal nanoparticles (AgNPs) were related to good biofilm eradication activity. In the current study, the capped AgNPs were spherical, with an average mean size in the range of (5.6–22.6 nm), which could facilitate antibacterial activity and provide more possible interactions with the bacterial surface. Additionally, it was thought that the ability of AgNPs to disrupt biofilm layers could be attributed to their severe inhibition of EPS matrix, deactivating the external protective layers of bacterial cell, as reported in [77]. Furthermore, a pH of the biofilm environment below 7 could change the negatively charged nanocomposites to positive ones, increasing electrostatic interactions with the bacterial surface [78]. Some studies illustrated that AgNPs tend to interact with molecular oxygen in an aqueous environment, thus giving more leached Ag^+^ ions and reactive oxygen species (ROS), which increased the toxic effects on the integrity of bacterial cell wall. Additionally, ROS can cause the total impairment of envelope-bound proteins, deoxyribonucleic acid (DNA), respiratory coenzymes, and antioxidants such as glutathione (GSH) [72]. Remarkably, these findings were consistent with the estimated antibiofilm activity (%) and scanning electron microscopy (SEM) images of AgNPs-treated *MRSA* (ATCC-43300) in the next section [79].

#### 3.2.5. Morphological Imaging of Treated *MRSA* (ATCC-43300)

**Figure 11:**
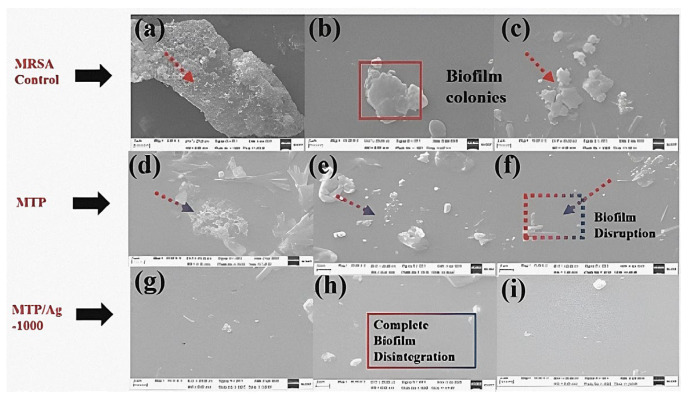
(**a**–**i**) SEM images showing the biofilm reduction of treated *MRSA* (ATCC-43300) by (100 µg/mL) of MTP and MTP/Ag-1000 on plasma-coated titanium surface at different magnifications.

**Figure 12:**
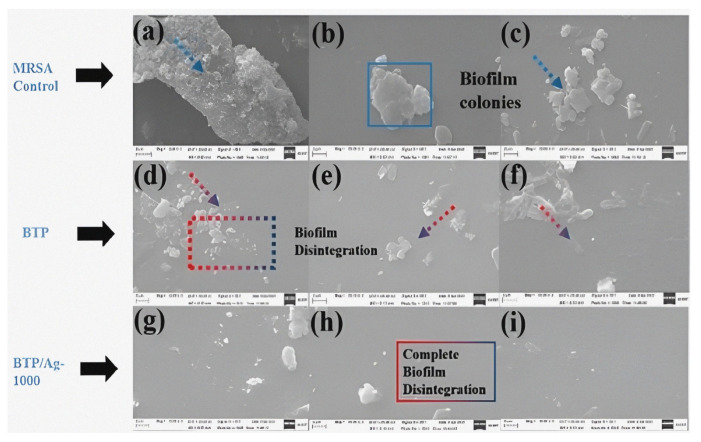
(**a**–**i**) SEM images showing the biofilm reduction of treated *MRSA* (ATCC-43300) by (100 µg/mL) of BTP and BTP/Ag-1000 on plasma-coated titanium surface at different magnification.

The biofilm inhibition of *MRSA* (ATCC-43300) was also qualitatively verified through scanning electron microscopy (SEM) after incubation by 100 µg/mL of raw compounds and their TP/Ag-1000. The two upper images (Figure 11 and Figure 12) represent the positively controlled (untreated) *MRSA* (ATCC-43300) surface on which aggregating grape-like colonies were developed to form the biofilm. On the contrary, low percentages of biofilm clusters were present on the surfaces of *MRSA* (ATCC-43300) after treatment with raw MTP and BTP, indicating that these compounds could prevent bacterial biofilm formation and induce cell death. Moreover, almost no biofilm matrices could be observed on MRSA treated with MTP and BTP decorated with AgNPs, validating the potent biofilm disintegration ability of TP/Ag NCs. In summary, these observations were in line with antibiofilm investigation results and other studies referring to the ability of synthesized AgNPs to severely interfere with cell wall (biofilm) components, thus hampering metabolic functions, and thereby, MRSA (ATCC-43300) cells are killed [79].

### 3.3. Molecular Docking Study

**Figure 13:**
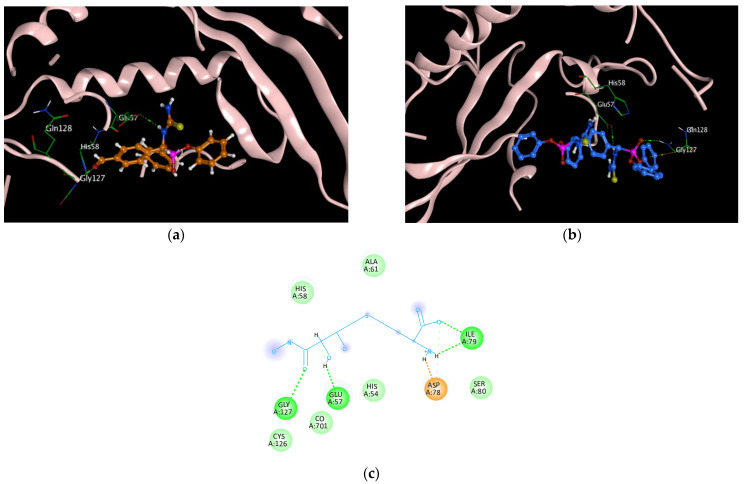
Chemical bonding involved in interaction between *bacillus subtilis* protein active site and (**a**) MTP compound via orange balls and sticks; (**b**) BTP compound in blue balls and sticks (whereas H-bonds have a green color and H-*pi* ones are yellow); and (**c**) the co-crystalized ligand 2D representation of the chemical interactions with the given protein, where H-bonds are in green while hydrophobic bonds are in orange.

**Figure 14:**
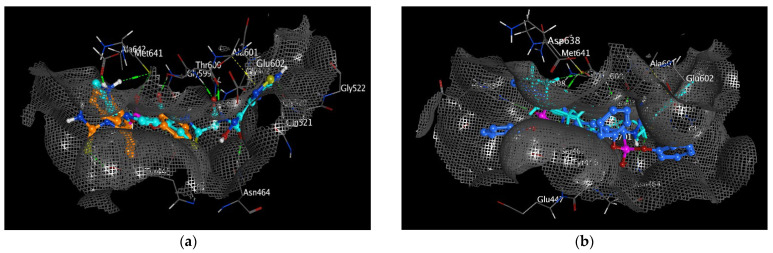

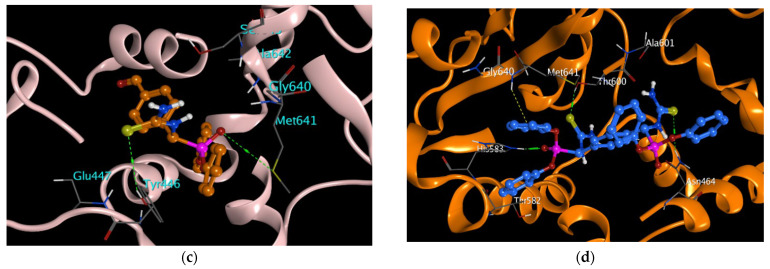
Surface of interaction at the *MRSA* protein active site showing the co-crystalized ligand in cyan balls and/or sticks complexed with (**a**) MTP compound (orange balls and sticks) and (**b**) BTP compound (blue balls and sticks). Chemical bonding involved in the interaction between the *MRSA* protein active site and (**c**) MTP compound (orange balls and sticks) and (**d**) BTP compound (blue balls and sticks), where H-bonds have a green color and H–pi bonds are yellow color.

**Figure 15:**
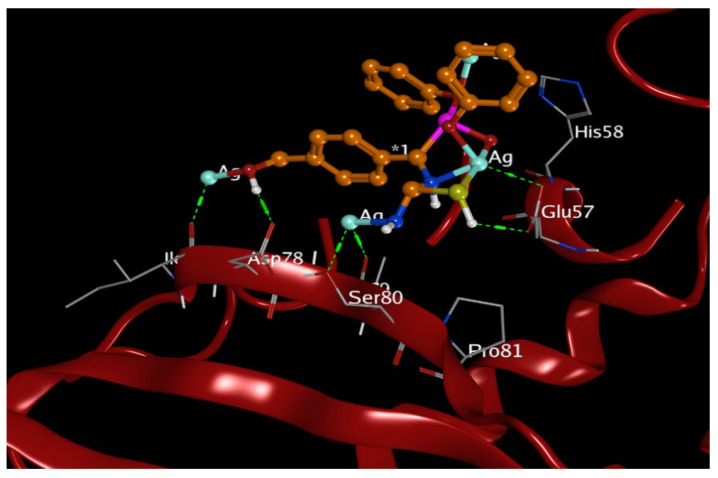
Site view of amino acids involved in interactions of MTP/Ag NC with *Bacillus subtilis* protein PDB-2FQT. *1 is the position of the co-crystalized ligand.

Molecular docking studies were conducted for the prepared TP compounds to investigate their binding affinity for Gram-positive (*MRSA* and *B. subtilis*) and Gram-negative (*P. aeruginosa* and *S. typhi*) bacterial proteins with respective codes of PDB: 4DKI, 2FQT, 5ZHN, and 3ZQE, in addition to confirming their illegibility as antibacterial agents with the experimental work. Furthermore, the docking results for the prepared raw compounds (MTP and BTP) in all downloaded proteins, along with type of chemical bonding and the amino acids involved in the interaction with the protein, were estimated. Additionally, values recorded for binding affinity and root mean square deviation (RMSD) were tabulated. There are different types of interactions, including hydrogen and hydrophobic H-*pi* interactions, with the binding sites of all the investigated proteins (Table 9). The best binding affinity for BTP was recorded with protein PDB (code: 2FQT) for *B. subtilis*, followed by protein PDB (code: 4DKI) for *MRSA*, with values of −7.9776 kcal/mol and −7.9074 kcal/mol, respectively. Amino acids involved in the interaction between BTP and the B. subtilis protein were GLY127 (H-acceptor, P=O), GLY127 (H-pi, H-Ar), and GLU57 (H-donor, >NH), which are the same amino acids involved in the interaction of the co-crystallized ligand and the protein (Figure 13). MTP also exhibited considerable binding interactions towards protein PDB (code: 4DKI) for MRSA then the 5ZHN of *P. aeruginosa*, with respective binding affinity values of −6.0126 and −5.976 kcal/mol. Moreover, the amino acids participating in the interactions with *MRSA* were MET641 (H-donor, P=O) and TYR446 (H-acceptor, C=S) (Figure 14), while TYR120 (H-donor) and GLU121 (H-donor) were contributed for *P. aeruginosa* interactions, consecutively (Table 9). It was noticed that the binding affinity and RMSD values were more promising for BTP than in MTP, which prompted our interest to incorporate AgNPs and evaluate the nano formulation effect on the antibacterial activity as conducted in the experimental work. Briefly, molecular modeling studies were performed for MTP-capped AgNPs based on the antibacterial progress that the biological results validated. Afterward, a noteworthy improvement against *B. subtilis* protein was observed, recording better binding affinity of −7.6668 kcal/mol, with a perfect fitting at the RMSD value of 1.2593 Å (Figure 15). Interestingly, these results were consistent with the experimental work, implying the considerable inhibition of AgNPs incorporated with MTP. As clearly demonstrated from the docking study, the superior efficacy of AgNPs could be attributed to the following: (a) The MTP/AgNP composites exerted Ag-acceptor bonds with both amino acids (SER80 and GLU57), along with the H-bonding interactions between raw MTP-capping compounds with amino acids of *B. subtilis* proteins (Figure 13). These binding interactions resulted in the formation of an Ag–protein complex, causing severe distortion in the elastic and geometric properties of the protein with several impairments to the biofilm [80]. (b) The inhibition of some biological enzymes, such as the S-ribosyl homocysteinase (LuxS), which is responsible for the communication system between *B. subtilis* cells via quorum sensing (QS) and thereby for biofilm growth [81,82]; thus, the (in silico) approach of TP-based analogues revealed their good stability profiles as potent antibacterial candidates.

It is pertinent to note that in this study, the structural and functional diversity of raw TP surfaces afforded highly tunable surface interactions and synergy, with AgNPs giving remarkable antibacterial activity. Additionally, the molecular docking studies were in good coherence with most experimental results for TPs and TP/Ag NCs. Based on the above considerations, some structural modifications may be applied in the future to exploit these compounds in various applications such as antibacterial textile fabrics, antibacterial food packaging films, and antibacterial thermoplastic polymer nanocomposites [83,84,85].

### 3.4. In Silico Pharmacokinetics Investigation

Absorption, distribution, metabolism, and excretion (ADME) analysis is very helpful in simplifying clinical trials, especially in the early stage of drug design. Intestinal absorption, skin sensitization, and oral bioavailability are absorption parameters considered in drug discovery [86]. An intestinal absorption score >30% indicates perfect absorbance [86,87]. As tabulated in Table 10, both compounds recorded intestinal absorption of more than 30%, with excellent absorbance rates of 92% and 69% achieved for MTP-AgNPs and BTP-AgNPs, respectively. A compound is known to have relatively low skin permeability if it has log K*p* > −2.5 [86]; however, the prepared TP/silver nanocomposites revealed good skin permeability, with permeability scores around −2.7cm/h. Compounds are considered to have high human colon adenocarcinoma (Caco-2) permeability when they record a Caco-2 value > 0.9 [86]; it measures the ability of a compound to cross a monolayer of Caco-2 cells, which are derived from human colon adenocarcinoma cells and have similar characteristics to small intestine epithelial cells. The currently investigated compounds revealed a moderate Caco2 score of 0.794 for MTP-AgNPs and very low score of −1.021 for BTP-AgNPs, which suggests that the compound may have poor absorption in the human small intestine. To investigate compounds’ distribution in silico, volume of distribution (VDss), blood–brain barrier (BBB) membrane permeability, and central nervous system (CNS) permeability, were assessed. VDss values disclosed distribution volumes with −0.573 and −1.411 log L/kg for MTP-AgNPs and BTP-AgNPs, respectively (Table 10). The log BB value for compounds will reflect low BBB if <−1, as reported. MTP-AgNPs expressed −1.404, while BTP-AgNPs disclosed a low permeability of the BBB membrane with a score of −2.836. Log permeability surface (PS) values for CNS permeability were −2.434 and −3.136 for MTP-AgNPs and BTP-AgNPs, respectively, and since low CNS permeability is reported if log PS is <−3 [86], MTP-AgNPs is considered to have promising CNS permeability while BTP-AgNPs shows CNS impermeability. Hepatic and renal clearance were used to examine the overall drug clearance. Total clearance calculates the drug concentration in the body utilizing the elimination rate. A compound’s excretion rate is demonstrated in log (mL/min/kg) (Table 10). The anticipated scores for ADME analysis are summarized (Table 10). In drug design, toxicity is a significant criterion which plays a remarkable role in the selection of sufficient drug candidates [88]. Regarding AMES toxicity, a positive test indicates a compound is mutagenic and carcinogenic [86,88]. Compound MTP-AgNPs has mutagen properties and hence the expected cytotoxicity; on the contrary, compound BTP-AgNPs recorded no AMES toxicity; however, both compounds revealed neither predicted hepatotoxicity nor skin allergic response. ERG inhibition (I and II) is a fundamental agent for toxicity analysis, in addition to it also including cardiotoxicity; hERG I was inhibited by both screened compounds. Toxicity against T. Pyriformis protozoa’s recorded IGC_50_ value was 0.335 and 0.285log μg/L for MTP-AgNPs and BTP-AgNPs, respectively. The anticipated scores are summarized below.

## 4. Conclusions

Two potent antibacterial agents were facilely synthesized in a one-pot green Kabachnik–Fields reaction and then exploited as reducing/capping surfaces for Ag^+^ ions affording TP/Ag NCs, the physicochemical properties of which were thoroughly investigated through FT-IR, (^1^H, ^13^C, and ^31^P)-NMR, elemental analysis, and TEM, XRD, and XPS analyses, respectively. The antibacterial properties of the as-prepared raw cores were studied via tuning different concentrations of silver nitrate, i.e., 100, 500, and 1000 mg L^−1^. Both raw Mono-TP and Bis-TP analogues proved to be bactericidal towards all isolates. In a dose-increasing manner, time–kill assay results manifested the record-breaking bactericidal effect of both MTP(BTP)/Ag-1000 towards all *MRSA* cells within 8 h. At high concentrations, the antifouling characteristics of evaluated compounds were influenced by the surface electrostatic repulsion with MRSA (ATCC-43300), while hydrophobicity played a critical role at lower concentrations. Targeting the biofilm of *MRSA* (ATCC-43300) was confirmed, with net activity of 79.5 and 83.6% for MTP/Ag-1000 and BTP/Ag-1000, progressively, at highest used concentration. Nonetheless, higher net antibiofilm activity was observed in BTP/Ag NCs; the simpler MTP structure tuned the surface work function with Ag^+^ ions, giving better colloidal AgNPs and a gradual improvement in the biofilm disruption % after the nanoloading. The docking results were in line with the experimental work, where the best results were estimated for raw BTP towards *B. subtilis*-2FQT protein, recording binding energy (BE) of −7.9776 Kcal/mol. Interestingly, the progress in BE of MTP/AgNPs to −7.6668 Kcal/mol could stem from the exerted Ag-acceptor bonds with both amino acids (SER80 and GLU57) of *B. subtilis*-2FQT protein. Further, in silico pharmacokinetics assessment was conducted as a step to predict the potential safety and toxicity of the TP/Ag NCs, which revealed AMES toxicity for one of the compounds with neither hepatic toxicity nor skin sensitization. In summary, this study has provided a good design for the synthesis and development of a new generation of nano-based aminophosphonate composites as promising antibacterial candidates, although their safety should be carefully evaluated for each specific application; further studies are needed to fully understand the toxicity mechanisms and the optimal conditions for their pharmaceutical application.

## Figures and Tables

**Table 1:** Antibacterial parameters of developed antibacterial agents—zone of inhibition (mm), minimum bactericidal concentration (MBC), minimum inhibitory concentration (MIC), and MBC/MIC ratio of compounds (mg/mL)—against *MRSA* and *MRSA* (ATCC-43300).

Compounds	ZOI	*MRSA*			*MRSA* (ATCC-43300)		
		MBC	MIC	MBC/MIC	MBC	MIC	MBC/MIC
MTP	28 ± 1.00	1.25	0.625	2(+)	2.5	1.25	2(+)
MTP/Ag-100	35 ± 1.00	0.3125	0.156	2(+)	0.625	0.3125	2(+)
MTP/Ag-500	44 ± 1.00	0.078	0.039	2(+)	0.078	0.039	2(+)
MTP/Ag-1000	49 ± 1.50	0.0006103	0.000305	2(+)	0.0006103	0.000305	2(+)
BTP	32 ± 1.00	0.625	0.3125	2(+)	2.5	1.25	2(+)
BTP/Ag-100	36 ± 1.00	0.3125	0.156	2(+)	0.625	0.3125	2(+)
BTP/Ag-500	40 ± 1.00	0.156	0.0781	2(+)	0.156	0.0781	2(+)
BTP/Ag-1000	52 ± 1.50	0.000305	0.0001525	2(+)	0.001220	0.0006103	2(+)
Ampicillin	14.77 ± 0.60	6.25	3.13	2(+)	6.25	1.56	4(+)
Ciprofloxacin	17.32 ± 0.60	3.13	0.78	4(+)	6.25	3.13	2(+)

**Table 2:** Antibacterial parameters of developed antibacterial agents—zone of inhibition (mm), minimum bactericidal concentration (MBC), minimum inhibitory concentration (MIC), and MBC/MIC ratio of compounds (mg/mL)—against *S. mutans* and *S. mutans* (ATCC-35668).

Compounds	ZOI	*S. mutans*			*S. mutans* (ATCC-35668)		
		MBC	MIC	MBC/MIC	MBC	MIC	MBC/MIC
MTP	30 ± 1.00	0.625	0.3125	2(+)	1.25	0.625	2(+)
MTP/Ag-100	36 ± 1.00	0.156	0.0781	2(+)	0.625	0.3125	2(+)
MTP/Ag-500	38 ± 1.00	0.03906	0.01953	2(+)	0.0781	0.03906	2(+)
MTP/Ag-1000	42 ± 1.00	0.000305	0.0001525	2(+)	0.001220	0.000305	2(+)
BTP	37 ± 0.60	0.3125	0.156	2(+)	0.625	0.3125	2(+)
BTP/Ag-100	40 ± 1.00	0.0781	0.03906	2(+)	0.156	0.0781	2(+)
BTP/Ag-500	43 ± 0.60	0.01953	0.009765	2(+)	0.0781	0.03906	2(+)
BTP/Ag-1000	49 ± 1.00	0.000305	0.0001525	2(+)	0.0006103	0.000305	2(+)
Ampicillin	12 ± 1.00	3.13	1.565	2(+)	6.25	3.13	2(+)
Ciprofloxacin	18.27 ± 0.60	6.25	1.56	4(+)	3.13	1.565	2(+)

**Table 3:** Antibacterial parameters of developed antibacterial agents—inhibition zone (mm), minimum bactericidal concentration (MBC), minimum inhibitory concentration (MIC) and MBC/MIC ratio of compounds (mg/mL)—against *B. subtilis* and *B. subtilis* (ATCC-6633).

Compounds	ZOI	*B. subtilis*			*B. subtilis* (ATCC-6633)		
		MBC	MIC	MBC/MIC	MBC	MIC	MBC/MIC
MTP	24 ± 1.00	0.625	0.3125	2(+)	1.25	0.625	2(+)
MTP/Ag-100	35 ± 1.00	0.03906	0.01953	2(+)	0.156	0.0781	2(+)
MTP/Ag-500	38 ± 1.00	0.00488	0.00244	2(+)	0.01953	0.009765	2(+)
MTP/Ag-1000	44 ± 1.00	0.0006103	0.000305	2(+)	0.00244	0.001220	2(+)
BTP	28 ± 1.00	0.156	0.0781	2(+)	0.625	0.3125	2(+)
BTP/Ag-100	36 ± 1.00	0.01953	0.009765	2(+)	0.03906	0.01953	2(+)
BTP/Ag-500	41 ± 1.00	0.009765	0.00488	2(+)	0.01953	0.009765	2(+)
BTP/Ag-1000	49 ± 1.00	0.000305	0.0001525	2(+)	0.001220	0.0006103	2(+)
Ampicillin	16.30 ± 0.6	8	4	2(+)	6.25	3.125	2(+)
Ciprofloxacin	18.32 ± 0.60	6.25	1.56	4(+)	3.13	0.78	4(+)

**Table 4:** Antibacterial parameters of developed antibacterial agents—zone of inhibition (mm), minimum bactericidal concentration (MBC), minimum inhibitory concentration (MIC) and MBC/MIC ratio of compounds (mg/mL)—against *P. aeruginosa* and *P. aeruginosa* (ATCC-27853).

Compounds	ZOI	*P. aeruginosa*			*P. aeruginosa* (ATCC-27853)		
		MBC	MIC	MBC/MIC	MBC	MIC	MBC/MIC
MTP	28 ± 1.00	1.25	0.625	2(+)	2.5	1.25	2(+)
MTP/Ag-100	34 ± 0.60	0.03906	0.01953	2(+)	0.156	0.0781	2(+)
MTP/Ag-500	39 ± 1.00	0.00488	0.00244	2(+)	0.01953	0.009765	2(+)
MTP/Ag-1000	43 ± 0.60	0.000305	0.0001525	2(+)	0.001220	0.0006103	2(+)
BTP	33 ± 0.60	0.156	0.0781	2(+)	0.625	0.3125	2(+)
BTP/Ag-100	39 ± 1.00	0.03906	0.01953	2(+)	0.156	0.0781	2(+)
BTP/Ag-500	43 ± 1.00	0.00488	0.00244	2(+)	0.01953	0.009765	2(+)
BTP/Ag-1000	47 ± 1.00	0.000305	0.0001525	2(+)	0.001220	0.0006103	2(+)
Ampicillin	8.7 ± 0.60	12.5	6.25	2(+)	6.25	1.56	4(+)
Ciprofloxacin	16.37 ± 0.60	6.25	1.56	4(+)	3.13	0.78	4(+)

**Table 5:** Antibacterial parameters of developed antibacterial agents—inhibition zone (mm), minimum bactericidal concentration (MBC), minimum inhibitory concentration (MIC) and MBC/MIC ratio of compounds (mg/mL)—against *S. typhi* and *S. typhi* (ATCC-6539).

Compounds	ZOI	*S. typhi*			*S. typhi* (ATCC-6539)		
		MBC	MIC	MBC/MIC	MBC	MIC	MBC/MIC
MTP	28 ± 1.00	0.625	0.3125	2(+)	1.25	0.625	2(+)
MTP/Ag-100	38 ± 1.00	0.03906	0.01953	2(+)	0.156	0.0781	2(+)
MTP/Ag-500	41 ± 1.00	0.00488	0.00244	2(+)	0.01953	0.009765	2(+)
MTP/Ag-1000	48 ± 1.00	0.000305	0.0001525	2(+)	0.001220	0.0006103	2(+)
BTP	35 ± 1.00	0.156	0.0781	2(+)	0.625	0.3125	2(+)
BTP/Ag-100	43 ± 1.00	0.01953	0.009765	2(+)	0.0781	0.03906	2(+)
BTP/Ag-500	48 ± 1.00	0.00244	0.001220	2(+)	0.009765	0.00488	2(+)
BTP/Ag-1000	54 ± 1.00	0.0001525	0.00007629	2(+)	0.0006103	0.000305	2(+)
Ampicillin	14.32 ± 0.60	3.13	1.565	2(+)	6.25	1.56	4(+)
Ciprofloxacin	18.32 ± 0.60	12.5	6.25	2(+)	3.13	0.78	4(+)

**Table 6:** Antibacterial parameters of developed antibacterial agents—zone of inhibition (mm), minimum bactericidal concentration (MBC), minimum inhibitory concentration (MIC), and MBC/MIC ratio of compounds (mg/mL)—against *S. marcescens* and *S. marcescens* (ATCC-13880).

Compounds	ZOI	*S. marcescens*			*S. marcescens* (ATCC-13880)		
		MBC	MIC	MBC/MIC	MBC	MIC	MBC/MIC
MTP	35 ± 1.00	1.25	0.625	2(+)	2.5	1.25	2(+)
MTP/Ag-100	41 ± 1.00	0.0781	0.03906	2(+)	0.3125	0.156	2(+)
MTP/Ag-500	45 ± 1.00	0.009765	0.00488	2(+)	0.01953	0.009765	2(+)
MTP/Ag-1000	49 ± 1.00	0.001220	0.0006103	2(+)	0.00244	0.00122	2(+)
BTP	38 ± 1.00	0.3125	0.156	2(+)	1.25	0.625	2(+)
BTP/Ag-100	44 ± 0.60	0.03906	0.01953	2(+)	0.156	0.0781	2(+)
BTP/Ag-500	47 ± 1.50	0.00244	0.001220	2(+)	0.01953	0.009765	2(+)
BTP/Ag-1000	54 ± 1.00	0.000305	0.0001525	2(+)	0.002244	0.001220	2(+)
Ampicillin	14.32 ± 0.60	6.25	3.125	2(+)	3.13	1.565	2(+)
Ciprofloxacin	14.50 ± 0.60	3.13	0.78	4(+)	6.25	1.56	4(+)

**Table 7:** Antiadhesion assay of tested compounds towards *MRSA* (ATCC-43300) based on quadruplicate results with standard deviation, (n = 4).

Concentration	MTP	MTP/Ag-100	MTP/Ag-500	MTP/Ag-1000	BTP	BTP/Ag-100	BTP/Ag-500	BTP/Ag-1000
10 mg/mL	11.8 ± 0.06	33.6 ± 0.11	50.2 ± 0.11	58.8 ± 0.08	25.4 ± 0.05	49.5 ± 0.22	55.4 ± 0.05	67.4 ± 0.11
5 mg/mL	5.3 ± 0.06	28.4 ± 0.08	41.9 ± 0.06	47.5 ± 0.13	19.8 ± 0.08	35.5 ± 0.11	49.8 ± 0.08	54.2 ± 0.11
2.5 mg/mL	3.8 ± 0.07	12.6 ± 0.09	24.7 ± 0.14	32.3 ± 0.12	8.8 ± 0.07	27.3 ± 0.07	33.8 ± 0.07	39.3 ± 0.09
1.25 mg/mL	1.7 ± 0.06	8.8 ± 0.06	16.4 ± 0.09	24.9 ± 0.09	3.4 ± 0.05	18.5 ± 0.07	23.4 ± 0.05	28.5 ± 0.09

**Table 8:** Anti-biofilm assay of tested compounds towards *MRSA* (ATCC-43300) (experiment evaluated based on quadruplicate results with standard deviation (n = 4)).

Concentration	MTP	MTP/Ag-100	MTP/Ag-500	MTP/Ag-1000	BTP	BTP/Ag-100	BTP/Ag-500	BTP/Ag-1000
10 mg/mL	50.2 ± 0.11	68.3 ± 0.08	72.7 ± 0.06	79.5 ± 0.02	62.9 ± 0.07	71.8 ± 0.06	77.4 ± 0.07	83.6 ± 0.11
5 mg/mL	41.9 ± 0.06	57.3 ± 0.08	68.6 ± 0.08	75.5 ± 0.11	58.8 ± 0.07	65.3 ± 0.06	71.9 ± 0.06	78.4 ± 0.08
2.5 mg/mL	24.7 ± 0.13	43.3 ± 0.05	52.8 ± 0.08	57.3 ± 0.07	43.9 ± 0.07	51.8 ± 0.07	61.4 ± 0.07	68.6 ± 0.09
1.25 mg/mL	16.4 ± 0.09	32.8 ± 0.06	34.7 ± 0.07	38.5 ± 0.07	34.6 ± 0.08	43.7 ± 0.06	46.4 ± 0.07	48.8 ± 0.06

**Table 9:** Docking results of MTP and BTP compounds with four different proteins of Gram-positive and negative bacterial strains.

Compound	Protein		BE (Kcal/mol)	RMSD (Å)	Amino Acid Interactions
	Bacterial Strains	PDB Code			
MTP	*MRSA*	4DKI	−6.0126	1.4454	MET641 (H-donor), TYR446(H-acceptor).
	*Bacillus*	2FQT	−5.562	1.979	GLU57(H-donor), GLY127(H-acceptor).
	*Pseudo*	5ZHN	−5.976	2.247	TYR120(H-donor), GLU121(H-donor).
	*Typhi*	3ZQE	−4.8091	1.7241	ARG58(H-acceptor).
BTP	*MRSA*	4DKI	−7.9074	1.7812	THR600(H-acceptor), HIS583(H-acceptor), ASN464(H-acceptor), GLY640 (H-*pi*).
	*Bacillus*	2FQT	−7.9776	1.8475	GLY127(H-acceptor), GLY127 (*pi*-H), GLU57 (H-donor)
	*Pseudo*	5ZHN	−5.7701	1.5838	SER (H-*pi*).
	*Typhi*	3ZQE	−6.7920	1.8609	ALA229(H-donor), TYR54 (*pi*-H).

**Table 10:** In silico ADMET assessment of the prepared TP/Ag NCs.

Model Name	Predicted Value for MTP-AgNPs	Predicted Value for BTP-AgNPs
Absorption		
Water solubility (log mol/L)	−4.889	−2.945
Caco-2 permeability (log Papp in 10^−6^ cm/s	0.794	−1.021
Intestinal absorption (human) (% Absorbed)	92.615	69.71
Skin Permeability (log Kp)	−2.738	−2.735
Distribution		
VDss (human)	−0.573	−1.411
Fraction unbound (human)	0	0.253
BBB permeability	−1.404	−2.836
CNS permeability	−2.434	−3.136
Metabolism		
CYP2D6 substrate	No	Yes
CYP3A4 substrate	Yes	Yes
CYP1A2 inhibitor	Yes	No
CYP2C19 inhibitor	Yes	Yes
CYP2C9 inhibitor	Yes	Yes
CYP2D6 inhibitor	No	No
CYP3A4 inhibition	Yes	Yes
Excretion		
Total Clearance (log mL/min/kg)	−0.555	−1.316
Renal OCT2 substrate (Yes/No)	No	No
Toxicity		
AMES toxicity	Yes	No
Max. tolerated dose (human) (log mg/kg/day)	0.145	0.412
hERG I inhibitor	No	No
hERG II inhibitor	Yes	Yes
Oral Rat Acute Toxicity (LD50) (mol/kg)	2.532	2.439
Hepatotoxicity	No	No
Skin Sensitization	No	No
*T. Pyriformis* toxicity (log μg/L)	0.335	0.285

## Data Availability

Not applicable.

## Supplementary Materials

The following supporting information can be downloaded at: https://www.mdpi.com/article/10.3390/pharmaceutics15061666/s1, Figure S1 (a,b) 1H-NMR spectra; (c,d) 13C-NMR spectra; and (e,f) 31P-NMR spectra of MTP and BTP derivatives, respectively, Figure S2. The element percentages of (a) MTP; and (b) BTP derivatives. Figure S3. The structural effect of MTP and BTP on AgNPs loading, Figure S4. Digital photos of MTP, BTP, and their Ag NCs at (100, 500, and 1000) mg L^−1^ displaying gradual changes in color with concentration increase of nanoloading, Figure S5. XPS spectra for (a) C 1s; (b) N 1s; (c) O 1s; (d) S 2p; (e) P 2p; and (f) Ag 3d in MTP/Ag500 with reference to MTP, Figure S6. Digital photos of inhibition zones developed by all antibacterial agents against MRSA compared to positive and negative controls. Figure S7. ZOI diameter percentage for treated MRSA by all studied compounds comparable to both references, Figure S8. Digital photos of inhibition zones developed by all antibacterial agents against S. mutans compared to positive and negative controls, Figure S9. Digital photos of inhibition zones developed by all antibacterial agents against B. subtilis compared to positive and negative controls, Figure S10. Digital photos of inhibition zones developed by all antibacterial agents against P. aeruginosa compared to positive and negative controls, Figure S11. Digital photos of inhibition zones developed by all antibacterial agents against S. typhi compared to positive and negative controls, Figure S12. Digital photos of inhibition zones developed by all antibacterial agents against S. marcescens compared to positive and negative controls, Figure S13. 3D-representation figure for the ZOI values of all antibacterial agents towards the treated Gram positive and negative strains.; Figure S14. The zeta potential of (a) MTP/Ag-1000; and (b) BTP/Ag-10000 respectively, Figure S15. Influence of MTP, and MTP/Ag NCs (100, 500, and 1000 mg Ag L^−1^ ) on the viability of MRSA (ATCC-43300) biofilm via crystal violet staining. The experiments were conducted with three duplicates, Figure S16. Influence of BTP, and BTP/Ag NCs (100, 500, and 1000 mg Ag L^−1^ ) on the viability of MRSA (ATCC-43300) biofilm via crystal violet staining. The experiments were conducted with three duplicates.; Table S1. The elemental analysis data of MTP and BTP conjugates, Table S2. The FT-IR data of MTP, BTP, and their AgNP dispersions in 100, 500, and 1000 mg Ag L^−1^, Table S3. XPS data along with the chemical assignments for each functional group in MTP/Ag-500 NC in comparison to AgNPs-free MTP, Table S4. Comparative ZOI and MIC values of studied antibacterial agents with some reported in literature (References [89,90,91,92,93,94,95,96,97,98,99,100,101] are cited in the Supplementary Materials).
