# Supplementary material for: Synthesis, Characterization, and Docking Study of Novel Thioureidophosphonate-Incorporated Silver Nanocomposites as Potent Antibacterial Agents
Source: Pharmaceutics. 2023 Jun 6;15(6):1666. doi: 10.3390/pharmaceutics15061666 (PMC10304937; doi:10.3390/pharmaceutics15061666)
Supplement: Supplementary file 1 [file pharmaceutics-15-01666-s001.zip › pharmaceutics-2417358-supplementary.pdf]

pharmaceutics

MDPI

Article

Synthesis, Characterization, and Docking Study of Novel Thioureidophosphonate-Incorporated Silver Nanocomposites as Potent Antibacterial Agents

Ahmed I. El-Tantawy [1,*], Elshaymaa I. Elmongy [2,*], Shimaa M. Elsaeed [3], Abdel Aleem H. Abdel Aleem [1], Reem Binsuwaidan [2], Wael H. Eisa [4], Ayah Usama Salman [5], Noura Elsayed Elharony [1] and Nour F. Attia [6]

1 Department of Chemistry, Faculty of Science, Menoufia University, Shibin El Kom 32511, Egypt
2 Department of Pharmaceutical Sciences, College of Pharmacy, Princess Nourah bint Abdulrahman University, P.O. Box 84428, Riyadh 11671, Saudi Arabia
3 Department of Analysis and Evaluation, Egyptian Petroleum Research Institute, Cairo 11727, Egypt
4 Spectroscopy Department, Physics Division, National Research Centre (NRC), Cairo 12622, Egypt
5 Department of Botany and Microbiology, Faculty of Science, Menoufia University, Shibin El Kom 32511, Egypt
6 Gas Analysis and Fire Safety Laboratory, Chemistry Division, National Institute for Standards, 136, Giza 12211, Egypt
* Correspondence: chemahmed293@gmail.com (A.I.E.-T.); eielmongy@pnu.edu.sa (E.I.E.)

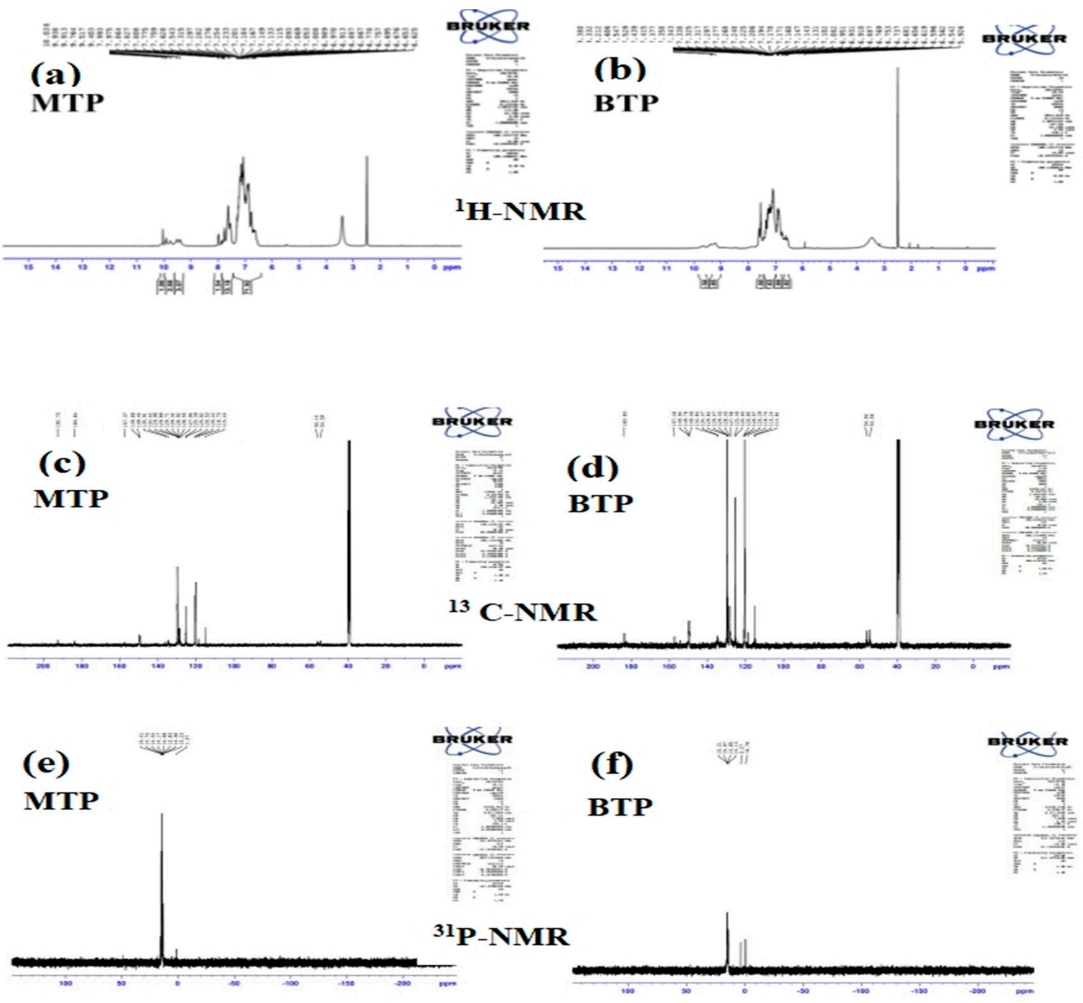

Figure S1. (a-b) ^{1}H-NMR spectra; (c-d) ^{13}C-NMR spectra; and (e-f) ^{31}P-NMR spectra of MTP and BTP derivatives, respectively.

Table S1. The elemental analysis data of MTP and BTP conjugates.

Sample	MTP						BTP					
	C	H	N	P	S	O	C	H	N	P	S	O
**Wt., (%)**	57.85	4.27	6.34	6.97	6.99	17.58	54.97	4.36	8.18	8.83	7.91	15.76
**mmol g^{-1}**	48.17	42.37	4.53	2.25	2.18	10.99	45.77	43.26	5.84	2.85	2.47	9.85
**Wt. T. (%)**	59.15	4.49	6.57	7.26	7.52	15.01	56.82	4.49	7.82	8.62	8.92	13.36
**Formula**	**$C_{21}H_{19}N_2O_4PS$**						**$C_{34}H_{32}N_4O_6P_2S_2$**					
**Mwt. (g mol^{-1})**	426.43						718.72					

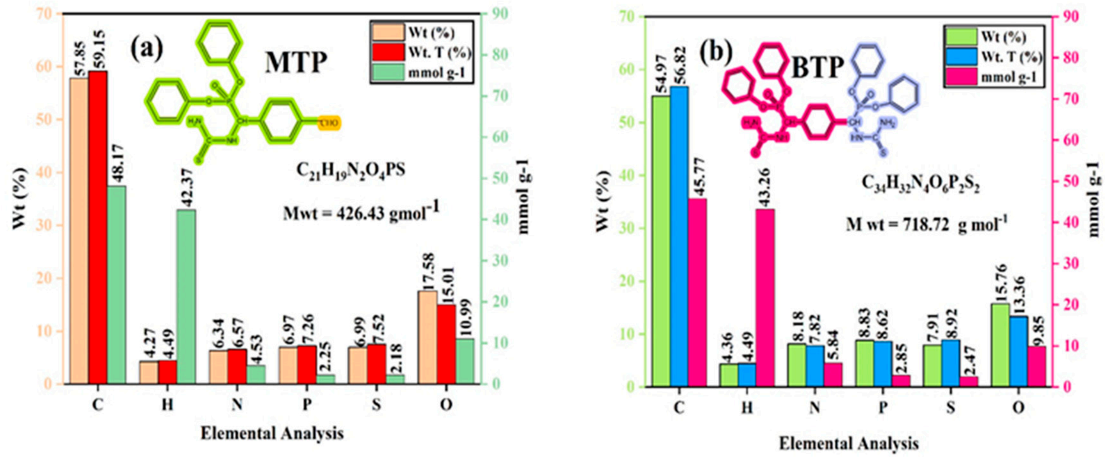

Figure S2. The element percentages of (a) MTP; and (b) BTP derivatives.

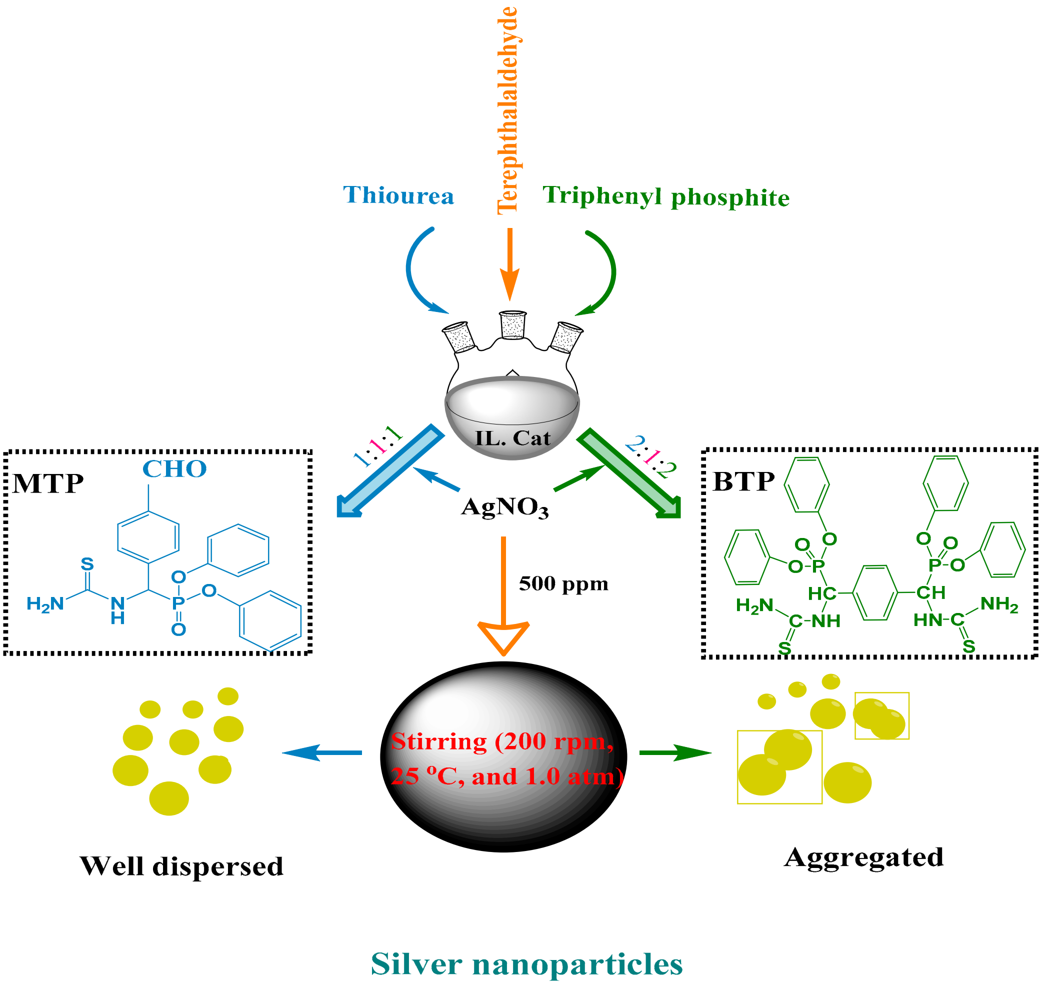

Figure S3. The structural effect of MTP and BTP on AgNPs loading.

pharmaceutics

MDPI

Table S2. The FT-IR data of MTP, BTP, and their AgNP dispersions in 100, 500, and 1000 mg Ag L^{-1}.

FT-IR (KBr method), υ cm^{-1}												
**Com.**	**>NH**	**-NH_2**	**Ar-H**	**C-H**	**-CHO**	**-NH_2**	**>C=C<**	**>C=S Asym.**	**P=O**	**P-O-C**	**P-C**	**C=S Sym.**
**MTP**	**3458**	**3311**	3058	**2895**	1698	1591	1525	1489	1195	945	761	685
**MTP/Ag 100**	**3465**	**3315**	3057	2897	1698	1591	1525	1488	1192	944	761	685
**MTP/Ag 500**	**3461**	**3304**	3058	2895	1699	1590	1525	1490	1196	945	762	686
**MTP/Ag 1000**	**3453**		3061	**2914**	1700	1593	1526	1488	1193	944	762	686
**BTP**	**3410**		3058	2896	----	1592	1527	1490	1196	948	762	688
**BTP/Ag 100**	**3306**		3061	2896	----	1594	1520	1486	1190	946	762	686
**BTP/Ag 500**	**3305**		3057	2896	----	1591	1528	1490	1194	947	762	687
**BTP/Ag 1000**	**3428**	**3320**	3057	2896	----	1595	----	1489	1195	948	763	687

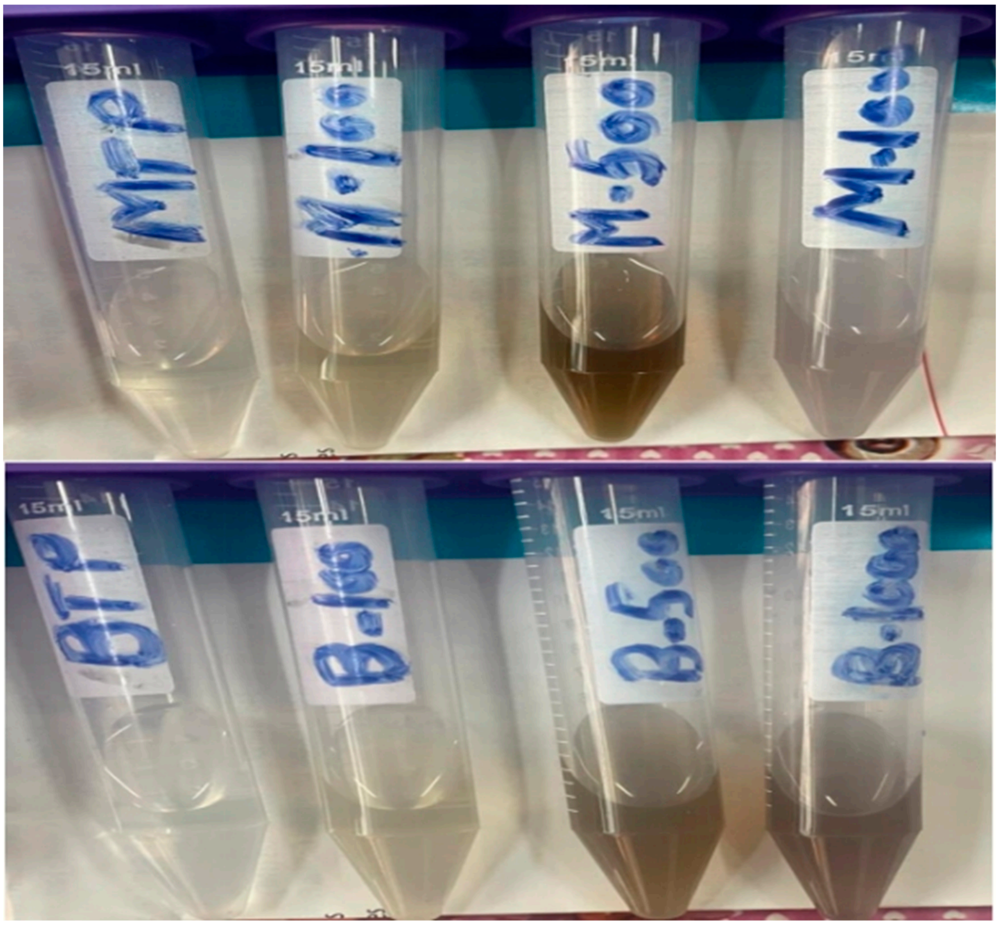

Figure S4. Digital photos of MTP, BTP, and their Ag NCs at (100, 500, and 1000) mg L^{-1} displaying gradual changes in color with concentration increase of nanoloading.

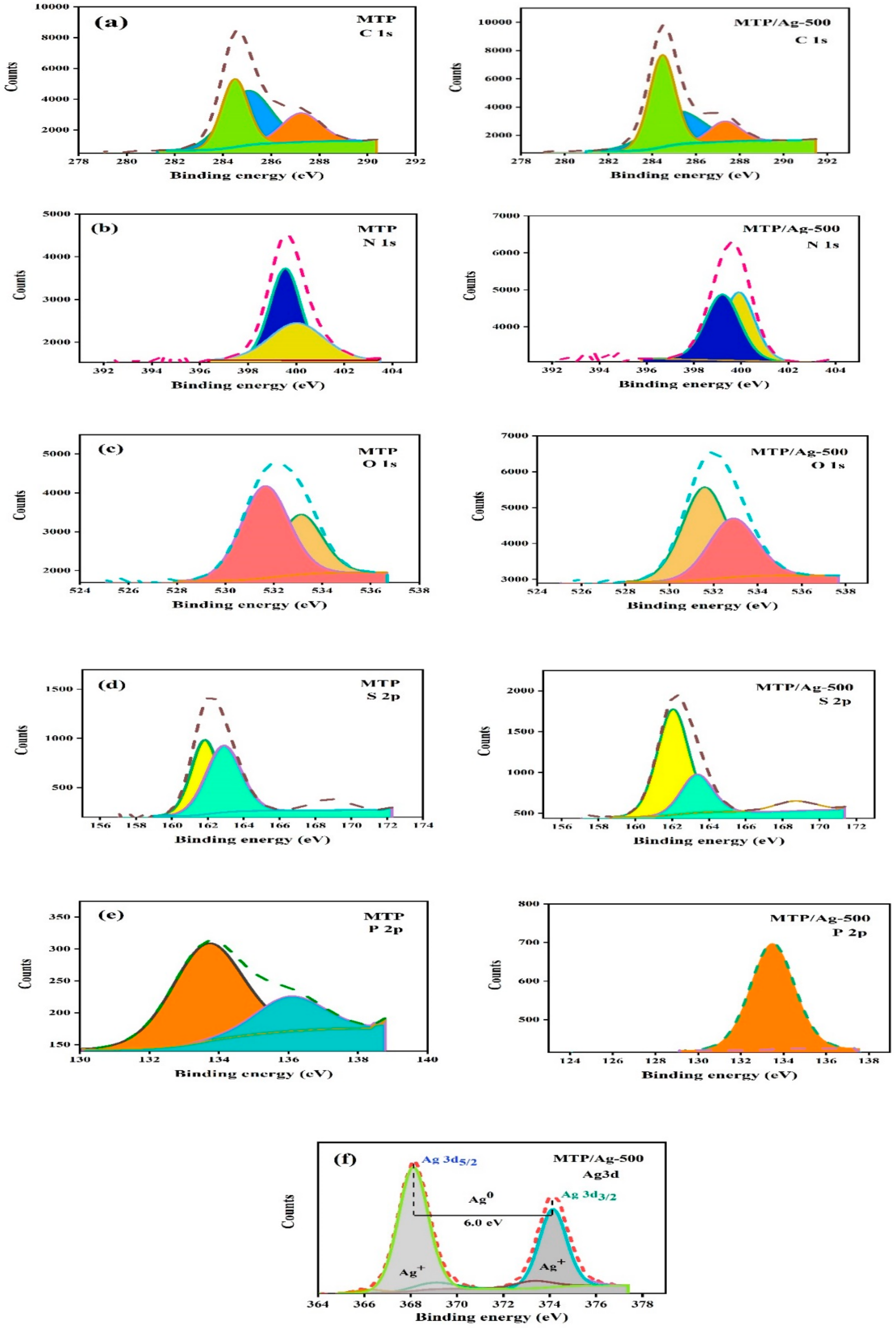

Figure S5. XPS spectra for (a) C 1s; (b) N 1s; (c) O 1s; (d) S 2p; (e) P 2p; and (f) Ag 3d in MTP/Ag-500 with reference to MTP core.

Table S3. XPS data along with the chemical assignments for each functional group in MTP/Ag-500 NC in comparison to AgNPs-free MTP.

	MTP			MTP/Ag-500			Chemical Assignments
	Peak BE	FWHM (eV)	Atomic %	Peak BE	FWHM (eV)	Atomic %	
C 1s	284.8	2.3	52.77	285.1	2.92	37.58	**C-H, C-C, C=C**
	284.5	1.31	27.04	284.46	1.4	49.36	**C-O, C-N, C-P, C=O**
	287.24	1.91	20.2	287.32	1.68	13.06	**π-π* sat, benzene rings, C=S**
N 1s	399.63	1.52	53.52	399.73	1.45	44.01	**C-N**
	399.75	2.71	46.48	399.56	2.53	55.99	**>NH /-NH_2 (amine)**
O 1s	531.64	2.31	62.6	531.58	2.37	59.21	**C-OH, P-O, C-O-C**
	533.11	2.12	37.4	532.88	2.57	40.79	**C=O, -OH (H_2O)**
P 2p	133.67	2.41	74.43	133.48	2.42	100	**$P2p_{1/2}$ (P-C, P-O)**
	136.05	2.3	25.57	---	----	----	**$P2p_{3/2}$ (P-O)**
S 2p	161.83	1.81	42.21	162.05	2.02	67.11	**C-S ($2p_{3/2}$)**
	162.89	2.32	48.72	163.35	2.03	24.55	**C-S ($2p_{1/2}$)**
	168.83	2.53	9.06	168.66	2.71	8.34	**C=S ($2p_{3/2}$)**
Ag 3d				368.11	1.63	54.88	**Ag^0 (Ag $5d_{5/2}$)**
				369.03	2.18	3.41	**Ag^+ (Ag $3d_{5/2}$: Ag-N/O)**
				374.14	1.48	37.40	**Ag^0 (Ag $5d_{3/2}$)**
				374.67	2.03	4.31	**Ag^+ (Ag $3d_{3/2}$: Ag-N/O)**

1. Antibacterial evaluation

The antibacterial activities of thioureidophosphonates (TPs) and their AgNP formulations were investigated against a series of Gram positive and negative bacteria using the agar well diffusion method. The initial inoculated colonies reached 1.5×10^8 CFU/mL, (0.5 McFarland scale) for all studied bacterial strains. The nutrient agar was poured in petri plates, sterilized, and then kept solidified. Wells in a diameter of 6 mm, were then made with the aid of a sterile cork borer. Afterwards, each well was filled with 0.5 mL of inoculated bacterial strain and 100 µL (10 mg/mL) of all tested compounds. Then, the plates placed in the refrigerator for 30 min allowing the DMSO dissolved compounds to diffuse into the agar, were put in the incubator for 18 h, at 37 °C. Moreover, the zone of inhibition (ZOI) diameters, including well diameter of 6 mm, were estimated with standard deviation values. Lastly, all antibacterial experiments were conducted in triplicates [1].

2. Investigation of bactericidal susceptibility

The minimum inhibitory concentration (MIC) values of all studied compounds and reference antibiotics (Ampicillin and Ciprofloxacin) were assessed against a series of clinical and their reference american type culture collection (ATCC) isolates of (*MRSA*, *S. mutans*, *B. subtilis*, *P. aeruginosa*, *S. typhi*, and *S. marcescens*), utilizing the microtiter broth dilution method [2]. Firstly, the evaluated compounds were individually dissolved in DMSO to a concentration of 20

mg/mL using 96-well microplates, then followed by a two folded serial dilution using Mueller–Hinton broth (MHB) through 36 dilutions from 10 mg/mL to 2.9103 × 10^{-10} mg/mL. Secondly, 100 µL was inoculated from the compound suspension (1.5×10^{8} CFU/mL) into each well. Then, the microtiter plates were incubated at 37°C for 24 h. Additionally, 40 µL of 0.4 mg/mL solution of INT was added to each well as an indicator of microbial growth at 37°C. Finally, the plates were incubated at 37°C for 30 min and the MIC values were visually determined. MIC is defined as the lowest concentration needed for complete inhibition of bacterial growth. In addition, MIC values were determined at least in duplicates and repeated to confirm activity. A negative control experiment was performed using only DMSO and MHB alone to ensure sterility. Furthermore, minimum bactericidal concentration (MBC) as a lowest sufficient concentration to kill almost the bacterial inoculations after 24h of incubation at 37°C, was recorded. Moreover, 10 µL was taken from the MIC obtained well and two wells above the MIC value well, were spread on MHA plates. After that, the number of colonies was counted after 18–24 h of incubation at 37°C. Then, the concentration of sample that produced <10 colonies was considered as MBC value. Each experiment was measured in triplicates [3]. Accordingly, all compounds were bactericidal with MBC/MIC ratio appropriate for time kill assay study. Meanwhile, around 1 × 10^{8} cells of *MRSA* culture were treated with all tested compounds and control. Then they were spotted on the TSA plates every 1 h and around 100 µL of both underwent serial dilution, followed by spreading on TSA plates prior to colony-forming unit (CFU) counting along with incubation for 24 h at 37 ℃ [4].

3. Bacterial culture preparation

Inoculate 3 to 5 pure colonies of *MRSA* (ATCC-43300) from the culture plate into 15 mL TSB with the bacterial revival in the shaker incubator at 200 rpm and at 37°C for 18 to 24 hours prior to being in their log phase of growth before the following assays. Furthermore, sterile TSB is used by autoclaving TSB at 121°C, 1.5 atm for 20 minutes.

4. Antiadhesion activity assay

Firstly, 50 µL of tested TP compounds and vancomycin were inoculated into designated 96-well microplate. DMSO was utilized as a negative control besides a diluent for the tested compounds. Then, *MRSA* (ATCC-43300) suspension of ~1 x 10^{8} CFU/ in 15 mL was prepared as an equivalent concentration to UV absorbance of (0.08 to 0.1), at λ_{max} = 600 nm. Moreover, a 0.5 McFarland standard was used to record the optimal bacterial suspension. Then, dilution was made to 100 times, giving 10^{6} CFU/mL bacterial suspension. Additionally, 50 µL of the diluted *MRSA* suspension was inoculated into tryptic soy broth (TSB) on a 96-well microplate, followed by sterilized distilled water addition and then the microplate was placed in the incubator at 37° C for 24 hours. Furthermore, TSB was slowly removed from the 96-well plate by decanting, in addition the plate was rinsed thrice with a sterilized distilled water and kept drying under the biosafety cabinet. Afterward, 100 µL of aqueous crystal violet (1% w/v) was dispensed into the test wells for 10-15 minutes for staining *MRSA* cells. Then, crystal violet was decanted into a sink and the test wells were rinsed 3 times with aseptic distilled water and kept drying. Finally, 30% (v/v) of glacial acetic acid was added in water to solubilize crystal violet and leave it standing for 15 minutes, without any visible residue of crystal violet in the test wells. Further, the UV absorbance readings for all wells were taken at λ_{max} = 570 nm (suggested range would be between 570 to 600 nm) [5].

Meanwhile, the antiadhesion activity (**%**) of evaluated formulations and vancomycin were determined using the following formula:

Antiadhesion activity (%) = 100 × ((Absorbance of Control - absorbance of test sample) /Absorbance of control).

5. Antibiofilm activity assay

The biofilm inhibition activity was established for TPs and their Ag NCs through the crystal violet method on a 96-well microplate. Firstly, 1.5 x 10^8 CFU/mL of *MRSA* (ATTC-43300) suspension was prepared and then diluted 1:100 times, affording 10^6 CFU/mL bacterial suspension. Thereafter, 100 µL of the diluted MRSA suspension was inoculated into TSB on a 96-well microplate, followed by adding a sterilized distilled water without vaporization and then the microplate was placed in the incubator at 37° C for 24 hours. After incubation, TSB was decanted, gently washed with aseptic phosphate buffer saline (PBS) for 3 times. Secondly, 100 µL of freshly prepared sterile TSB broth, 10 mg/mL of tested compounds dissolved in DMSO, and 100 mg/mL of positive control Vancomycin hydrochloride (VAN) were added in the whole system and then placed in the incubator at 37°C for 18 to 24 hours. Further, the TSB was decanted, in addition the microplate was rinsed with double distilled water and left for drying under biosafety cabinet. Subsequently, 100 µL of aqueous crystal violet (1% w/v) was poured into the test wells to stain the bacterial cell walls for (10-15) min. Afterwards, the crystal violet was decanted, and the test wells were 3 times rinsed with double distilled water till drying. Then, 30% (v/v) glacial acetic acid was added in water to solubilize crystal violet, leaving it standing for 15 minutes **[6].** Finally, the UV absorbance readings of each well at λ_{max} = 570 nm were taken via the microplate reader spectrophotometer, indicating the biofilm eradication ability in (%) for each evaluated compound along with Vancomycin (VAN), as observed in the equation below.
Antibiofilm activity (%) = 100 × ((Absorbance of control - Absorbance of test sample)/Absorbance of control).

6. Scanning electron microscopy (SEM)

SEM imaging was performed to evaluate the morphological effects on the *MRSA* (ATCC-43300) surface treated by TP compounds. To prepare the untreated and treated *MRSA* control, the glutaraldehyde fixation method was utilized. Moreover, the *MRSA* control were placed in a shaker incubator at 37 °C for 24 h. Then, they were collected through centrifugation and prefixed with a (2.5%) glutaraldehyde solution at 4 °C for 24 h. Thereafter, the *MRSA* cells were rinsed with PBS and post-fixed with 100 % acetone. Then, *MRSA* cells were sputtered with gold giving a layer 20 nm thick and the sample were investigated by SEM (JSM 6390 LA, JEOL, Tokyo, Japan) at a 15 KV accelerating voltage [7].

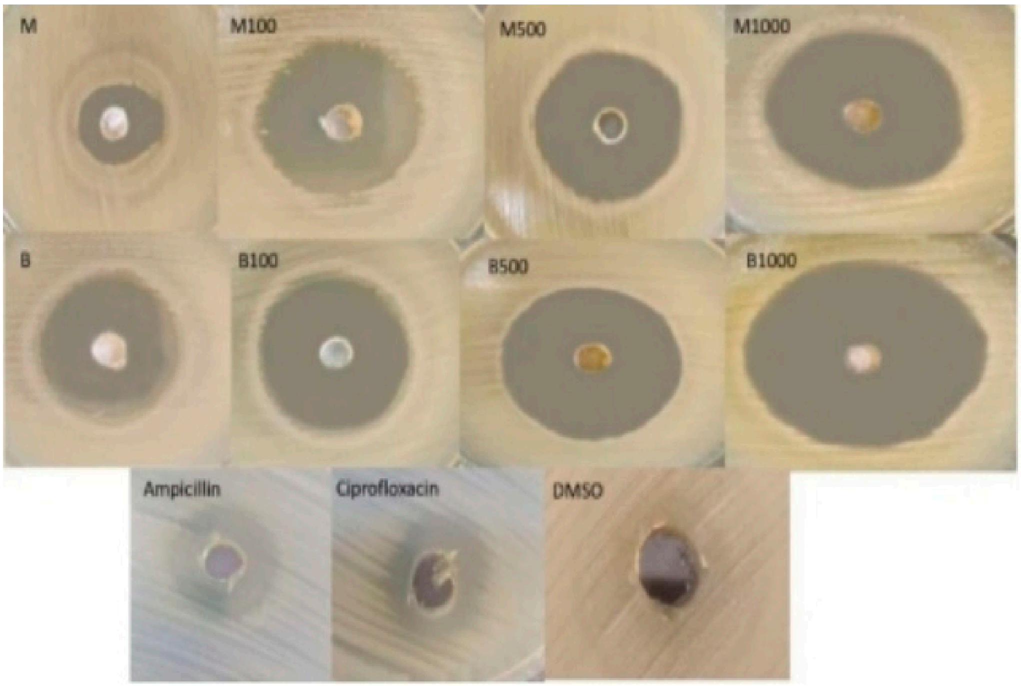

Figure S6. Digital photos of inhibition zones developed by all antibacterial agents against *MRSA* compared to positive and negative controls.

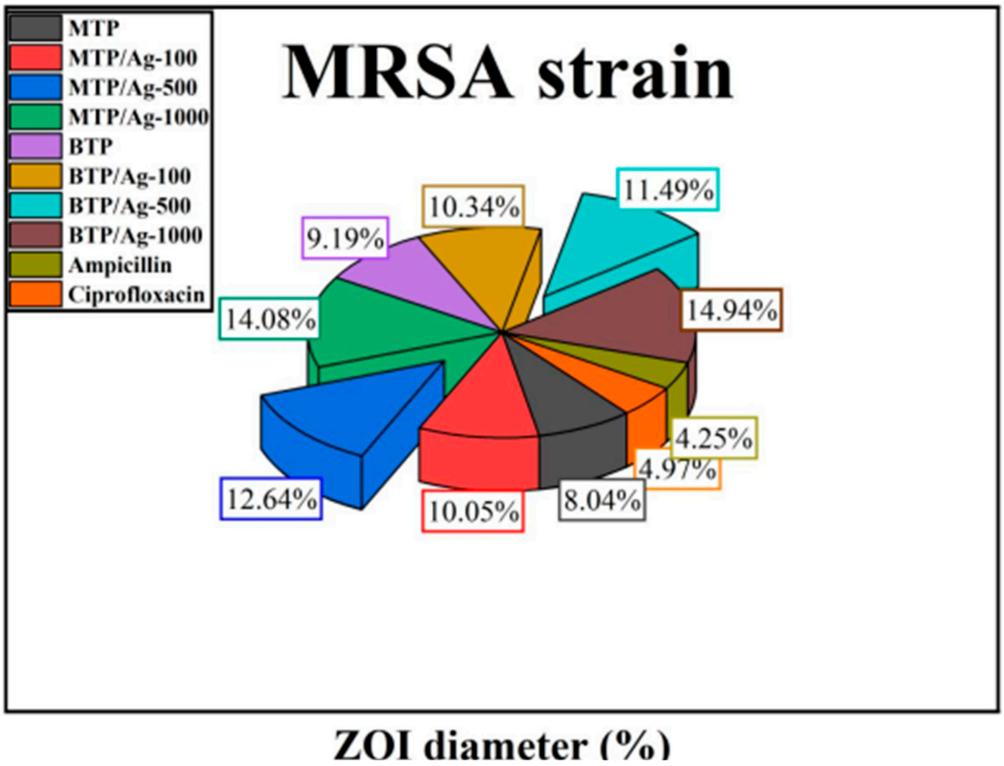

Figure S7. ZOI diameter percentage for treated *MRSA* by all studied compounds comparable to both references.

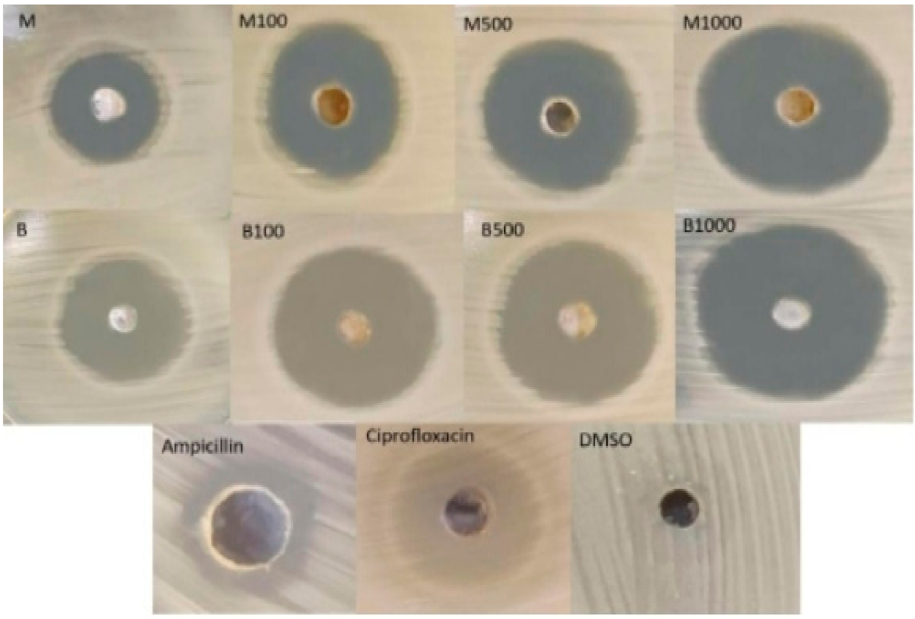

Figure S8. Digital photos of inhibition zones developed by all antibacterial agents against *S. mutans* compared to positive and negative controls.

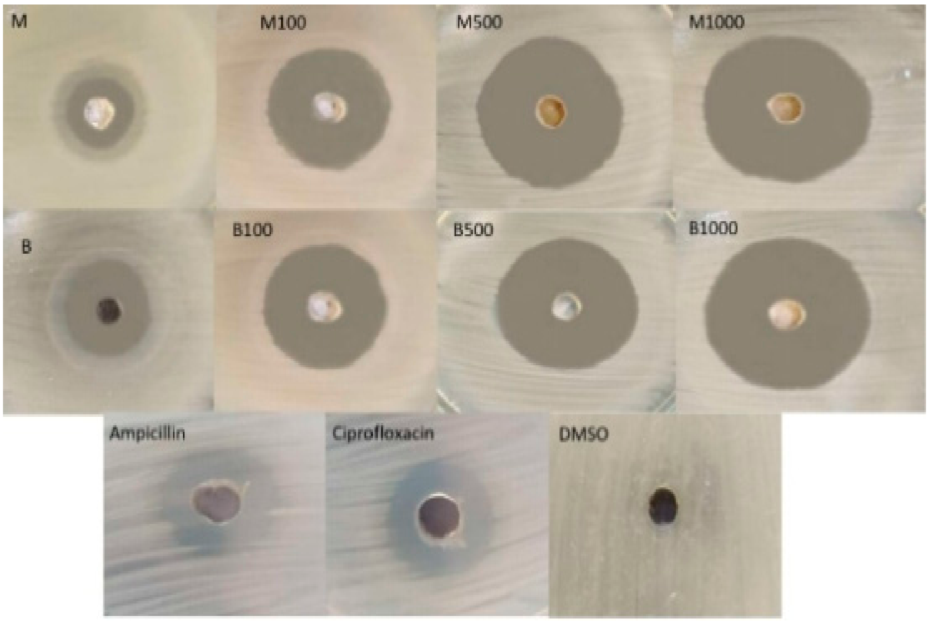

Figure S9. Digital photos of inhibition zones developed by all antibacterial agents against *B. subtilis* compared to positive and negative controls.

pharmaceutics

MDPI

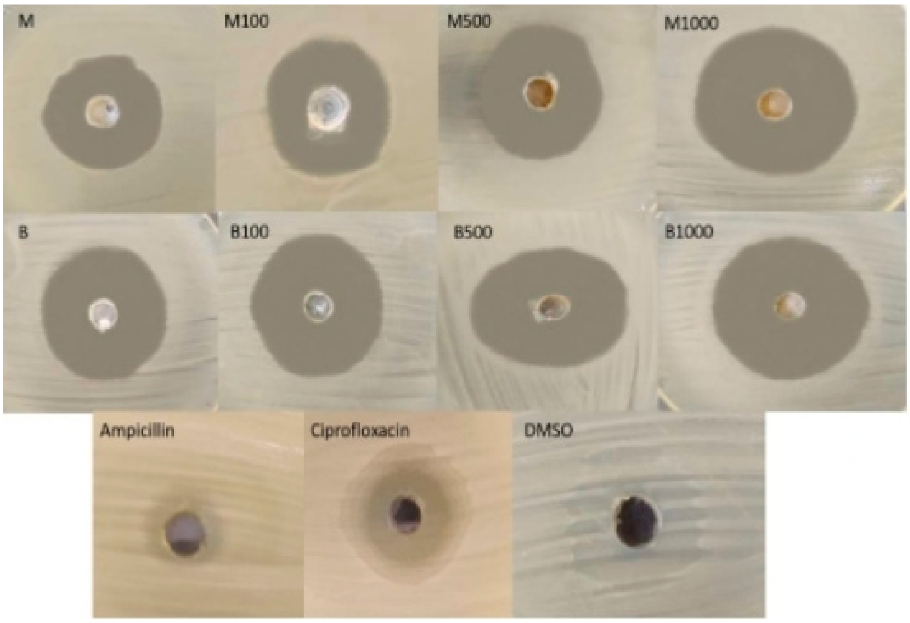

Figure S10. Digital photos of inhibition zones developed by all antibacterial agents against *P. aeruginosa* compared to positive and negative controls.

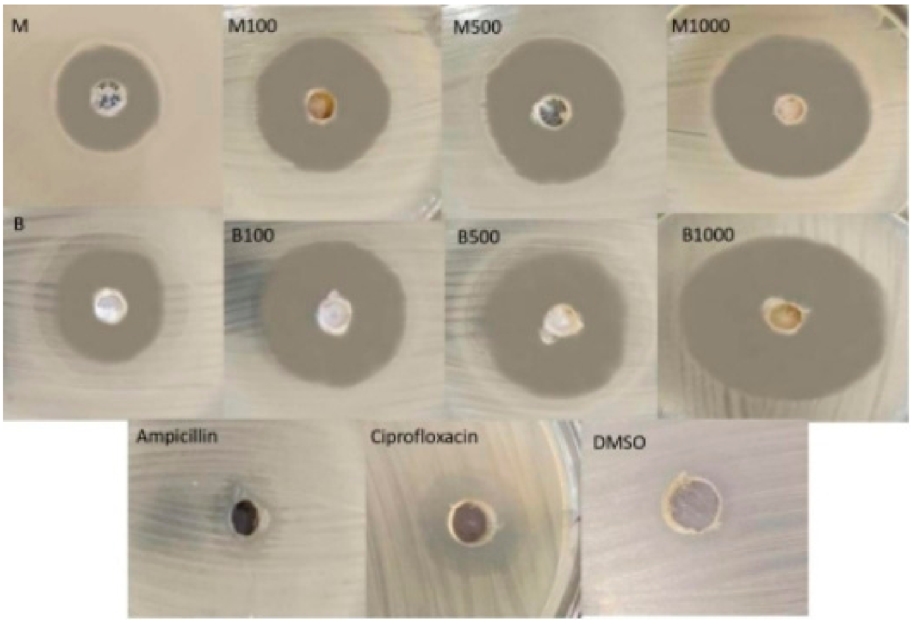

Figure S11. Digital photos of inhibition zones developed by all antibacterial agents against *S. typhi* compared to positive and negative controls.

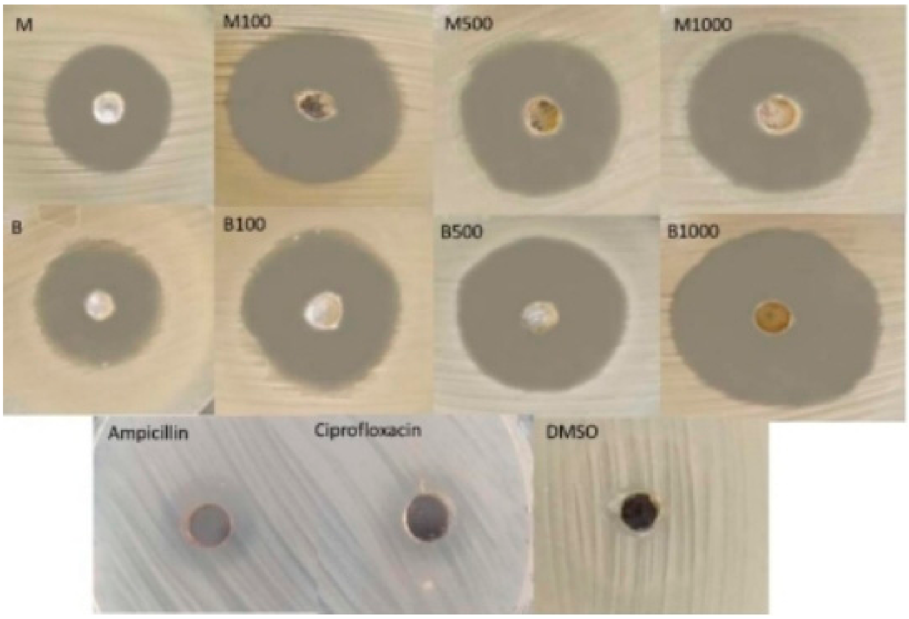

Figure S12. Digital photos of inhibition zones developed by all antibacterial agents against *S. marcescens* compared to positive and negative controls.

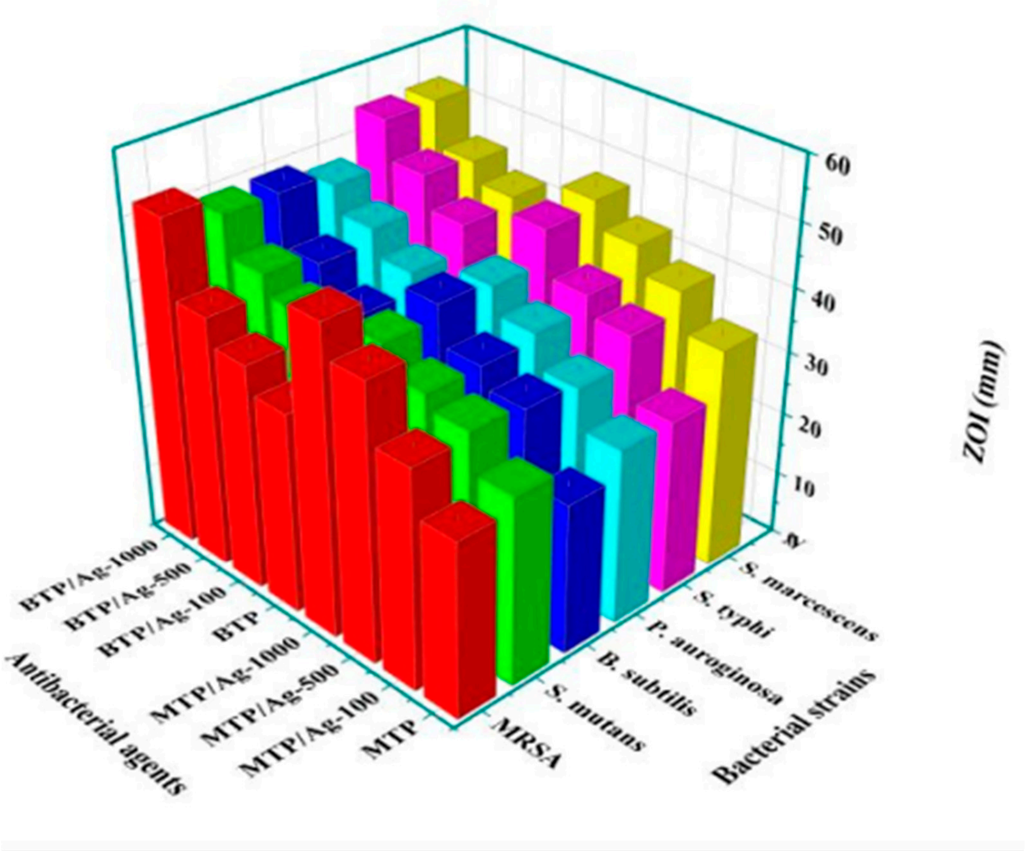

Figure S13. 3D-representation figure for the ZOI values of all antibacterial agents towards the treated Gram positive and negative strains.

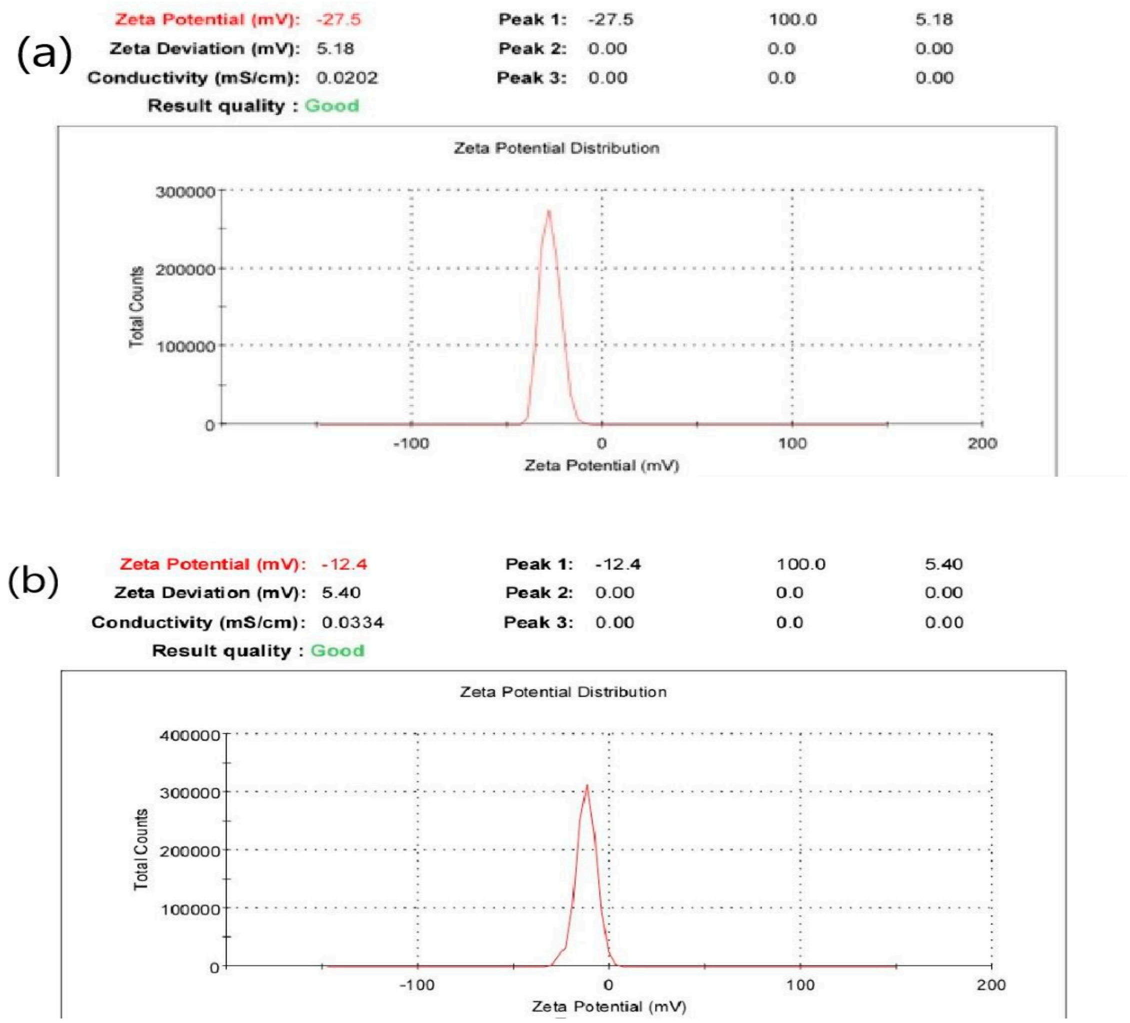

Figure S14. The zeta potential of (a) MTP/Ag-1000; and (b) BTP/Ag-10000 respectively.

pharmaceutics

Table S4. Comparative ZOI and MIC values of studied antibacterial agents with some reported in literature.

Sample Code	*MRSA*	*S. mutans*	*B. subtilis*	*P. aeruginosa*	*S. typhi*	*S. marcescens*	Ref
**Amaranthus spinosus L. Extract/AgNPs**	(17.0)[a] (0.1)[b]	----	----	(21.3)[a] (0.1)[b]	----	----	**[8]**
**Zingiber zerumbet/AgNPs**	(14.66)[a] (0.00312)[b]	(18.83)[a] (0.0125)[b]	----	----	----	----	**[9]**
**MTP/Ag-500**	(44.0)[a] (0.039)[b]	(38.0)[a] (0.01953)[b]	(38.0)[a] (0.00244)[b]	(39.0)[a] (0.00244)[b]	(41.0)[a] (0.00244)[b]	(45.0)[a] (0.00488)[b]	**This work**
**Lipopeptide /AgNPs**	----	----	(13)[a] (0.015625)[b]	(10)[a] (0.015625)[b]	----	----	**[10]**
**Grapefruit peel extract/AgNPs**	(12.7)[a] (0.0132)[b]	----	----	(14)[a] (0.05278)[b]	(12)[a] (0.05278)[b]	----	**[11]**
**Nostoc/AgNPs**	(18)[a] (0.9)[b]	(14.7)[a] (1.2)[b]	----	----	(14.8)[a] (0.9)[b]	----	**[12]**
**Glycolic acid/AgNPs**	(17.0)[a] (0.8)[b]	----	----	(17.0)[a] (0.8)[b]	(17.0)[a] (0.8)[b]	(12.0)[a] (0.8)[b]	**[13]**
**BTP/Ag-500**	(40.0)[a] (0.0781)[b]	(43.0)[a] (0.00976)[b]	(41.0)[a] (0.00488)[b]	(43.0)[a] (0.00244)[b]	(48.0)[a] (0.00122)[b]	(47.0)[a] (0.00122)[b]	**This work**

a = Zone of inhibition (ZOI) in mm, **b** = Minimum inhibitory concentration (MIC) in mg/mL.

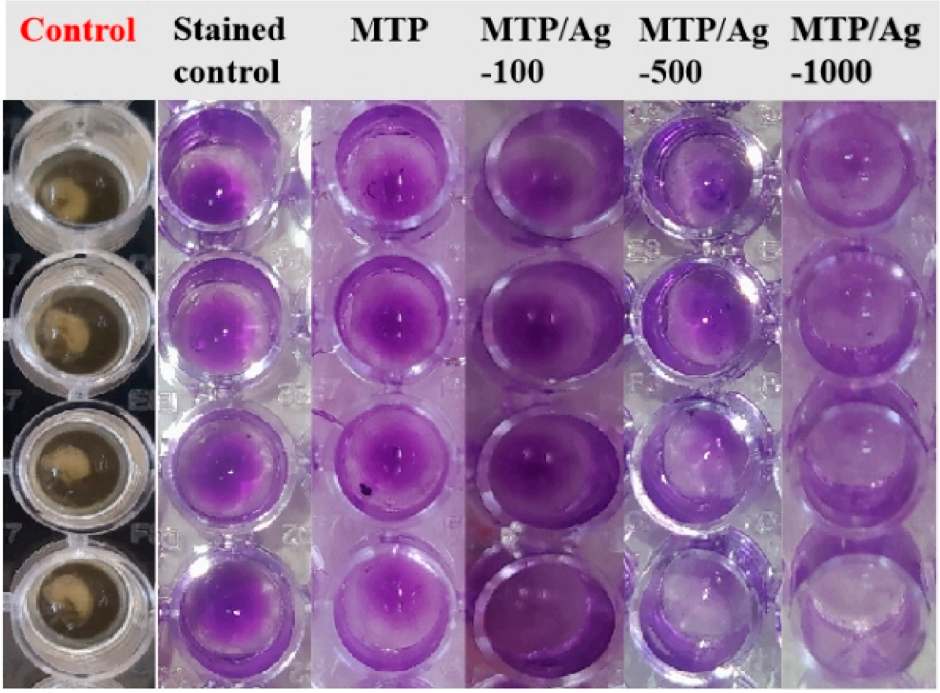

Figure S15. Influence of MTP, and MTP/Ag NCs (100, 500, and 1000 mg Ag L^{-1}) on the viability of *MRSA* (ATCC-43300) biofilm via crystal violet staining. The experiments were conducted with three duplicates.

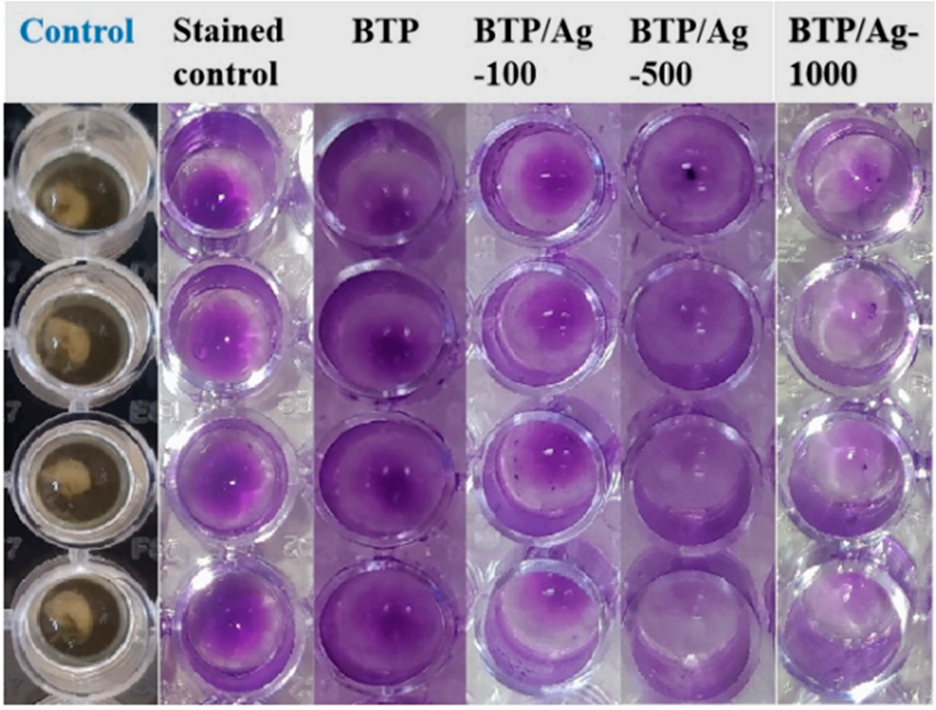

Figure S16. Influence of BTP, and BTP/Ag NCs (100, 500, and 1000 mg Ag L^{-1}) on the viability of *MRSA* (ATCC-43300) biofilm via crystal violet staining. The experiments were conducted with three duplicates.

References

1. Daoud, A., et al., *Assessment of polyphenol composition, antioxidant and antimicrobial properties of various extracts of Date Palm Pollen (DPP) from two Tunisian cultivars.* 2019. **12**(8): p. 3075-3086.

2. Eloff, J.N.J.P.m., *A sensitive and quick microplate method to determine the minimal inhibitory concentration of plant extracts for bacteria.* 1998. **64**(08): p. 711-713.

3. Ozturk, S., S.J.W.J.o.M. Ercisli, and Biotechnology, *Chemical composition and in vitro antibacterial activity of Seseli libanotis.* 2006. **22**: p. 261-265.

4. Poonacha, N., et al., *Efficient killing of planktonic and biofilm-embedded coagulase-negative staphylococci by bactericidal protein P128.* 2017. **61**(8): p. e00457-17.

5. Kemung, H.M., et al., *An optimized anti-adherence and anti-biofilm assay: Case study of zinc oxide nanoparticles versus MRSA biofilm.* 2020. **3**(1).

6. O'Toole, G.A.J.J., *Microtiter dish biofilm formation assay.* 2011(47): p. e2437.

7. Badger-Emeka, L.I., P.M. Emeka, and H.I.M.J.A.S. Ibrahim, *A Molecular Insight into the Synergistic Mechanism of Nigella sativa (Black Cumin) with β-Lactam Antibiotics against Clinical Isolates of Methicillin-Resistant Staphylococcus aureus.* 2021. **11**(7): p. 3206.

8. R, P. and D. Sebastian, *Characterization of Green Synthesized Antibacterial Silver Nanoparticles from Amaranthus spinosus L. Extract.* BioNanoScience, 2022. **12**(2): p. 502-511.

9. Ramzan, M., et al., *Synthesis of Silver Nanoparticles from Extracts of Wild Ginger (Zingiber zerumbet) with Antibacterial Activity against Selective Multidrug Resistant Oral Bacteria.* Molecules, 2022. **27**(6).

10. Bezza, F.A., S.M. Tichapondwa, and E.M.N. Chirwa, *Synthesis of biosurfactant stabilized silver nanoparticles, characterization and their potential application for bactericidal purposes.* Journal of Hazardous Materials, 2020. **393**: p. 122319.

11. Alsakhawy, S.A., et al., *Antibacterial Activity of Silver Nanoparticles Phytosynthesized by Citrus Fruit Peel Extracts.* BioNanoScience, 2022. **12**(4): p. 1106-1115.

12. Hamida, R.S., et al., *Lethal Mechanisms of Nostoc-Synthesized Silver Nanoparticles Against Different Pathogenic Bacteria.* Int J Nanomedicine, 2020. **15**: p. 10499-10517.

13. Kumar, M., et al., *Glycolic acid functionalized silver nanoparticles: A novel approach towards generation of effective antibacterial agent against skin infections.* Journal of Drug Delivery Science and Technology, 2020. **60**: p. 102074.
